# Oncogenic KRAS Regulates Tumor Cell Signaling via Stromal Reciprocation

**DOI:** 10.1016/j.cell.2016.03.029

**Published:** 2016-05-05

**Authors:** Christopher J. Tape, Stephanie Ling, Maria Dimitriadi, Kelly M. McMahon, Jonathan D. Worboys, Hui Sun Leong, Ida C. Norrie, Crispin J. Miller, George Poulogiannis, Douglas A. Lauffenburger, Claus Jørgensen

**Affiliations:** 1 The Institute of Cancer Research, 237 Fulham Road, London SW3 6JB, UK; 2 Department of Biological Engineering, Massachusetts Institute of Technology, Cambridge, MA 02139, USA; 3 Cancer Research UK Manchester Institute, University of Manchester, Wilmslow Road, Manchester M20 4BX, UK

## Abstract

Oncogenic mutations regulate signaling within both tumor cells and adjacent stromal cells. Here, we show that oncogenic KRAS (KRAS^G12D^) also regulates tumor cell signaling via stromal cells. By combining cell-specific proteome labeling with multivariate phosphoproteomics, we analyzed heterocellular KRAS^G12D^ signaling in pancreatic ductal adenocarcinoma (PDA) cells. Tumor cell KRAS^G12D^ engages heterotypic fibroblasts, which subsequently instigate reciprocal signaling in the tumor cells. Reciprocal signaling employs additional kinases and doubles the number of regulated signaling nodes from cell-autonomous KRAS^G12D^. Consequently, reciprocal KRAS^G12D^ produces a tumor cell phosphoproteome and total proteome that is distinct from cell-autonomous KRAS^G12D^ alone. Reciprocal signaling regulates tumor cell proliferation and apoptosis and increases mitochondrial capacity via an IGF1R/AXL-AKT axis. These results demonstrate that oncogene signaling should be viewed as a heterocellular process and that our existing cell-autonomous perspective underrepresents the extent of oncogene signaling in cancer.

**Video Abstract:**

## Highlights

• KRAS^G12D^ establishes a reciprocal signaling axis via heterotypic stromal cells
• Reciprocal signaling further regulates tumor cell signaling downstream of KRAS^G12D^
• Reciprocal signaling regulates tumor cell behavior via AXL/IGF1R-AKT
• Heterocellularity expands tumor cell signaling beyond cell-autonomous pathways

## Graphical Abstract

**Figure:**
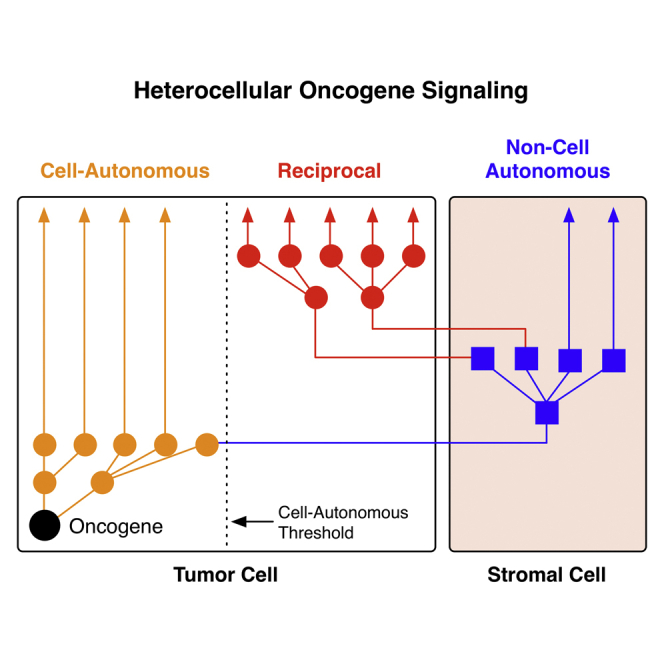

## Teaser

Cell-specific proteome labeling reveals that oncogenic KRAS stimulates stromal cells to initiate reciprocal signaling back to pancreatic tumor cells, thereby enabling signaling capacity beyond the traditionally studied cell-autonomous pathways.

## Introduction

Solid cancers are heterocellular systems containing both tumor cells and stromal cells. Coercion of stromal cells by tumor cell oncogenes profoundly impacts cancer biology (Friedl and Alexander, 2011, Quail and Joyce, 2013) and aberrant tumor-stroma signaling regulates many hallmarks of cancer (Hanahan and Weinberg, 2011). While individual oncogene-driven regulators of tumor-stroma signaling have been identified, the propagation of oncogene-dependent signals throughout a heterocellular system is poorly understood. Consequently, our perspective of oncogenic signaling is biased toward how oncogenes regulate tumor cells in isolation (Kolch et al., 2015).

In a heterocellular cancer, tumor cell oncogenes drive aberrant signaling both within tumor cells (cell-autonomous signaling) and adjacent stromal cells (non-cell-autonomous signaling) (Croce, 2008, Egeblad et al., 2010). As different cell types process signals via distinct pathways (Miller-Jensen et al., 2007), heterocellular systems (containing different cell types) theoretically provide increased signal processing capacity over homocellular systems (containing a single cell type). By extension, oncogene-dependent signaling can theoretically engage additional signaling pathways in a heterocellular system when compared to a homocellular system. However, to what extent activated stromal cells reciprocally regulate tumor cells beyond cell-autonomous signaling is not well understood.

We hypothesized that the expanded signaling capacity provided by stromal heterocellularity allows oncogenes to establish a differential reciprocal signaling state in tumor cells. To test this hypothesis, we studied oncogenic KRAS (KRAS^G12D^) signaling in pancreatic ductal adenocarcinoma (PDA). KRAS is one of the most frequently activated oncogenic drivers in cancer (Pylayeva-Gupta et al., 2011) and is mutated in >90% of PDA tumor cells (Almoguera et al., 1988). PDA is an extremely heterocellular malignancy—composed of mutated tumor cells, stromal fibroblasts, endothelial cells, and immune cells (Neesse et al., 2011). Crucially, the gross stromal pancreatic stellate cell (PSC) expansion observed in the PDA microenvironment is non-cell-autonomously controlled by tumor cell KRAS^G12D^ in vivo (Collins et al., 2012, Ying et al., 2012). As a result, understanding the heterocellular signaling consequences of KRAS^G12D^ is essential to comprehend PDA tumor biology.

Comprehensive analysis of tumor-stroma signaling requires concurrent measurement of cell-specific phosphorylation events. Recent advances in proteome labeling now permit cell-specific phosphoproteome analysis in heterocellular systems (Gauthier et al., 2013, Tape et al., 2014a). Furthermore, advances in proteomic multiplexing enable deep multivariate phospho-signaling analysis (McAlister et al., 2012, Tape et al., 2014b).

Here, we combine cell-specific proteome labeling, multivariate phosphoproteomics, and inducible oncogenic mutations to describe KRAS^G12D^ cell-autonomous, non-cell-autonomous, and reciprocal signaling across a heterocellular system. This study reveals KRAS^G12D^ uniquely regulates tumor cells via heterotypic stromal cells. By exploiting heterocellularity, reciprocal signaling enables KRAS^G12D^ to engage oncogenic signaling pathways beyond those regulated in a cell-autonomous manner. Expansion of KRAS^G12D^ signaling via stromal reciprocation suggests oncogenic communication should be viewed as a heterocellular process.

## Results

### Tumor Cell KRAS^G12D^ Non-cell-autonomously Regulates Stromal Cells

**Figure 1:**
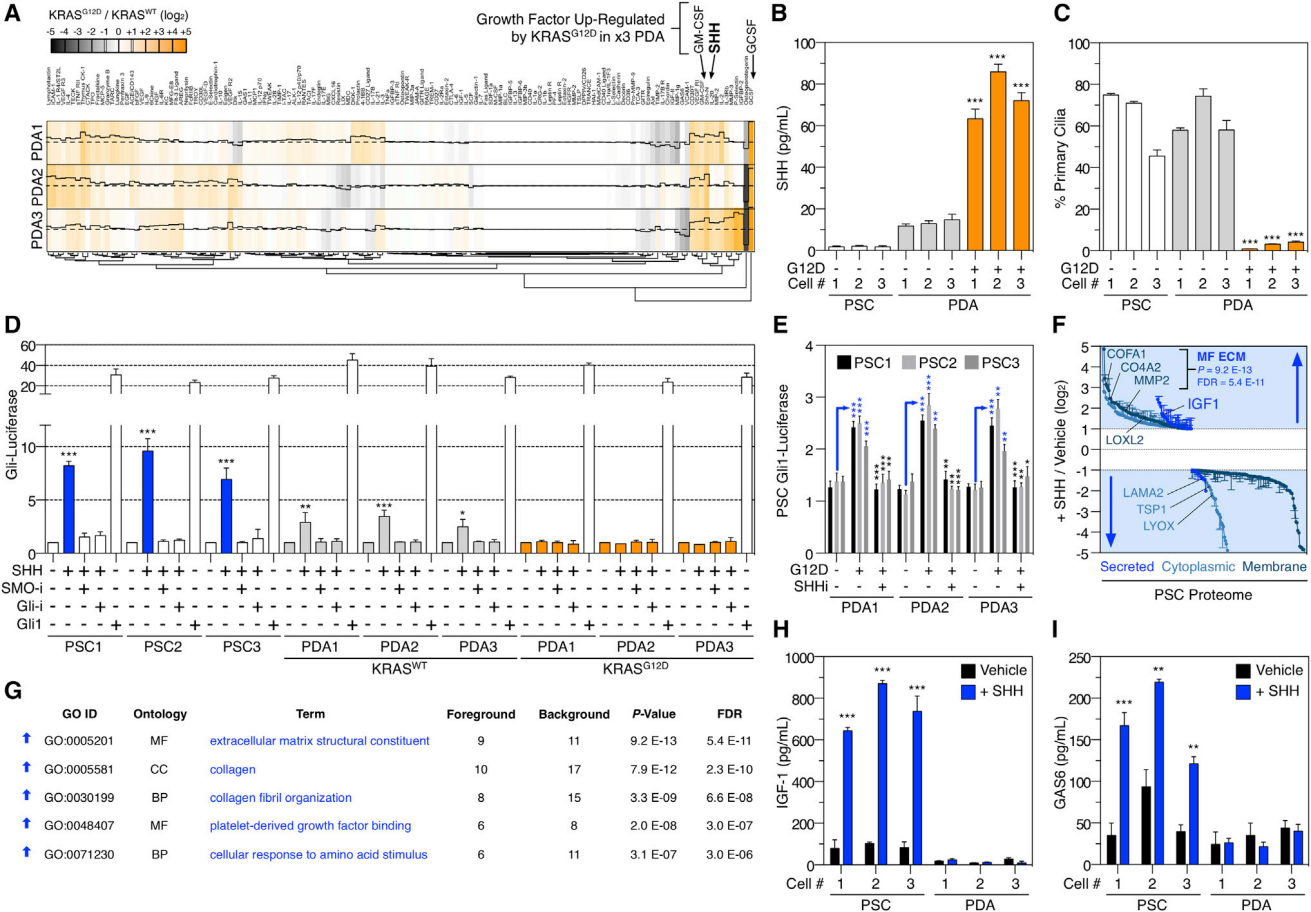
Tumor Cell KRAS^G12D^ Non-cell-autonomously Regulates PSCs (A) Soluble growth factor/cytokine/receptor array of conditioned media from three iKRAS PDA cell isolations (KRAS^G12D^/KRAS^WT^) (hierarchical clustering). KRAS^G12D^ increases GM-CSF, GCSF, and SHH protein secretion. (B) SHH ELISA of PDA and PSC conditioned media. PSC do not secrete SHH, whereas KRAS^G12D^ induces SHH secretion from PDA tumor cells (two-tailed t test) (n = 3). ^∗^p < 0.05, ^∗∗^p < 0.01, ^∗∗∗^p < 0.001. (C) High-content imaging primary cilia quantification (via acetylated tubulin) for all cells (48 hr) (n = 3). PSCs and KRAS^WT^ PDA cells possess primary cilia, whereas KRAS^G12D^ do not; t test: ^∗^p < 0.05, ^∗∗^p < 0.01, ^∗∗∗^p < 0.001. (D) PSCs and PDA cells (KRAS^G12D^ and KRAS^WT^) transfected with a Gli1-luciferase reporter stimulated with SHH for 48 hr ± Smoothened (SMO-i) or Gli (Gli-i) inhibitors. Ligand-dependent SHH signaling (via canonical SMO and Gli activity) is only observed in PSCs and KRAS^WT^ PDA cells (n = 3). ^∗^p < 0.05, ^∗∗^p < 0.01, ^∗∗∗^p < 0.001. (E) PSCs transfected with Gli1-luciferase reporter co-cultured with PDA cells ± SHH inhibitory antibody (SHHi). PDA KRAS^G12D^ secreted SHH initiates non-cell-autonomous signaling in PSCs. RLU fold-difference versus PSC+Gli1-luciferase in mono-culture (n = 3) (blue = stimulation, black = inhibition). ^∗^p < 0.05, ^∗∗^p < 0.01, ^∗∗∗^p < 0.001. (F) PSC cytoplasmic, membrane, and secreted proteomes regulated by SHH (48 hr). (G) DAVID GO-enrichment analysis of SHH non-cell-autonomously regulated PSC proteome (p < E−06). (H and I) SHH upregulates IGF-1 and GAS6 protein in PSCs, but not in KRAS^G12D^ PDA cells. ^∗^p < 0.05, ^∗∗^p < 0.01, ^∗∗∗^p < 0.001. See also Figure S1 and Data S1.

To investigate how KRAS^G12D^ supports heterocellular communication, we first analyzed tumor cell-secreted signals (using PDA tumor cells containing an endogenous doxycycline inducible KRAS^G12D^) (Collins et al., 2012, Ying et al., 2012). Measuring 144 growth factors, cytokines, and receptors across three unique PDA isolations, we observed that KRAS^G12D^ increased secretion of GM-CSF, GCSF cytokines, and the growth morphogen sonic hedgehog (SHH) (Figure 1A). As SHH regulates pancreatic myofibroblast expansion (Collins et al., 2012, Fendrich et al., 2011, Thayer et al., 2003, Tian et al., 2009, Yauch et al., 2008), and ablation of SHH signaling reduces PDA tumor stroma in vivo (Lee et al., 2014, Olive et al., 2009, Rhim et al., 2014), we focused on understanding the trans-cellular signaling consequences of SHH.

As previously established, KRAS^G12D^ simultaneously induces SHH secretion (Collins et al., 2012, Lauth et al., 2010) (Figure 1B) and disrupts primary cilium in PDA cells (Figure 1C). Concordantly, PSCs and KRAS^WT^ PDA cells transduce canonical SHH signaling (via SMO-GLI), while KRAS^G12D^ cells do not (Figure 1D). This enables KRAS^G12D^ PDA cells to non-cell-autonomously signal to PSCs via SHH, while remaining insensitive to autocrine SHH (Figure 1E).

**Figure S1:**
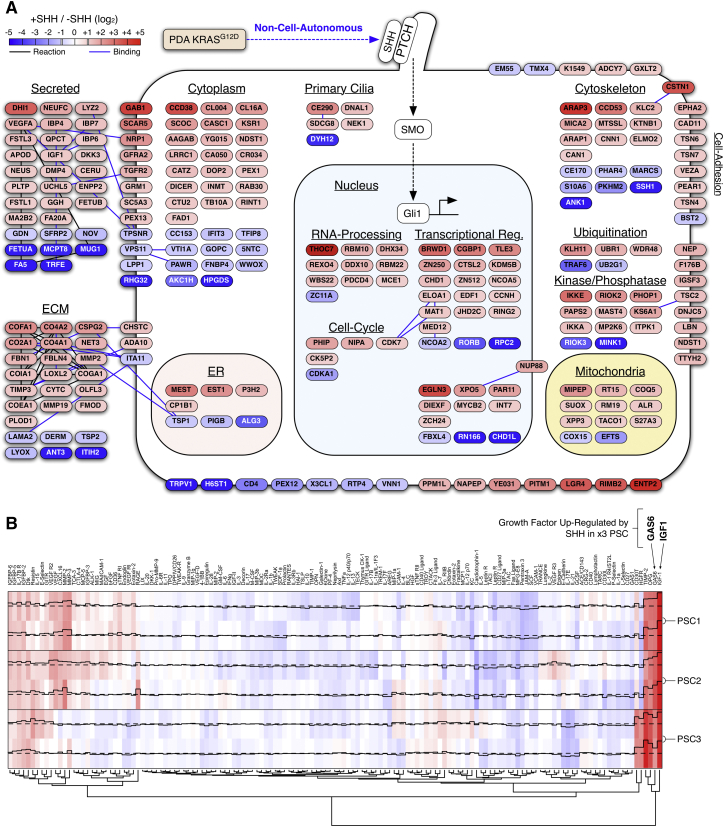
SHH Regulates Cytoplasmic, Membrane, and Secreted PSC Proteomes, Related to Figure 1 (A) Cellular heat map of regulated proteins from the experiment described in Figure 1F (Uniprot annotation). SHH stimulation of PSCs results in widespread differential regulation of secreted signaling molecules, cell-adhesion membrane proteins, components of the extracellular matrix (ECM), cytoplasmic molecules and nuclear proteins. String annotations for ‘Reaction’ and ‘Binding’ relationships are shown. (B) Soluble growth-factor and cytokine antibody array of conditioned media from PSCs stimulated with SHH or vehicle control (48 hr). SHH upregulates GAS6 and IGF1 across all three PSC isolations.

Quantitative proteomic analysis revealed SHH induces widespread changes across the cytoplasmic, membrane, and secreted PSC proteome (Figures 1F, 1G, and S1A; Data S1). SHH upregulates multiple extracellular matrix components (collagens, MMPs, fibrillin-1, LOX)—suggesting KRAS^G12D^ controls PDA desmoplasia via SHH-activated PSCs. Notably, SHH also upregulates IGF1 and GAS6 across multiple PSC isolations but not in PDA cells (Figures 1H, 1I, and S1B). Since IGF1 and GAS6 are growth factors capable of activating the receptor tyrosine kinases (RTKs) IGF1R and AXL, respectively, this suggests that SHH-activation alters the intercellular signaling potential of PSCs.

These results demonstrate KRAS^G12D^ non-cell-autonomously communicates with stromal cells via SHH-SMO-GLI and renders tumor cells insensitive to autocrine SHH. Moreover, KRAS^G12D^ achieves a unique signaling output (e.g., production of ECM, IGF1, and GAS6) via stromal cells that is distinct from that produced by tumor cell KRAS^G12D^ alone.

### KRAS^G12D^ Regulates Distinct Cell-Autonomous Signaling

**Figure 2:**
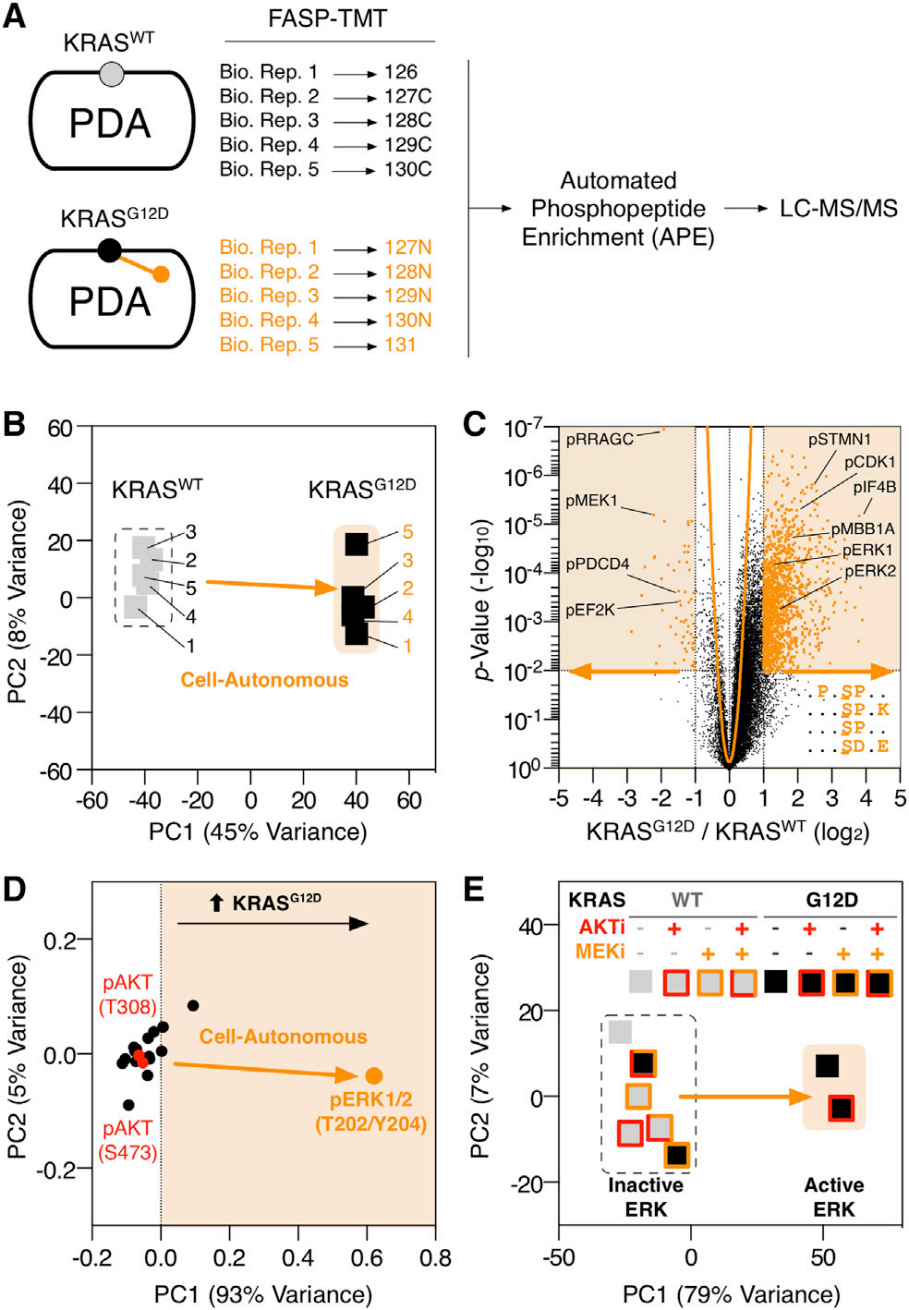
Cell-Autonomous KRAS^G12D^ Phosphoproteome (A) KRAS^WT^ and KRAS^G12D^ PDA cell lysates were isobarically labeled with tandem-mass tags (TMT) (126–131 mass-to-charge ratio [m/z]), mixed, and subjected to automatic phosphopeptide enrichment (APE) (n = 5). TMT-phosphopeptides were analyzed by high-resolution LC-MS/MS and normalized to total protein level changes. (B) KRAS^WT^ and KRAS^G12D^ phosphoproteomes cluster in PCA space. (C) Statistical regulation of the PDA KRAS^G12D^ cell-autonomous phosphoproteome (n = 5, two-tailed t test, Gaussian regression). Cell-autonomous enriched phospho-motifs shown. (D) PDA cell-autonomous regulation of 18 intracellular signaling nodes following KRAS^G12D^ induction across 48 hr (n = 3) in PCA space. (E) KRAS^WT^ and KRAS^G12D^ PDA cells treated ±MEK and AKT inhibitors analyzed by multivariate phosphoproteomics. KRAS^G12D^ cell-autonomous PDA phosphoproteomic state requires active MEK and is independent of AKT activity. See also Figures S2, S3, and Data S1.

**Figure S2:**
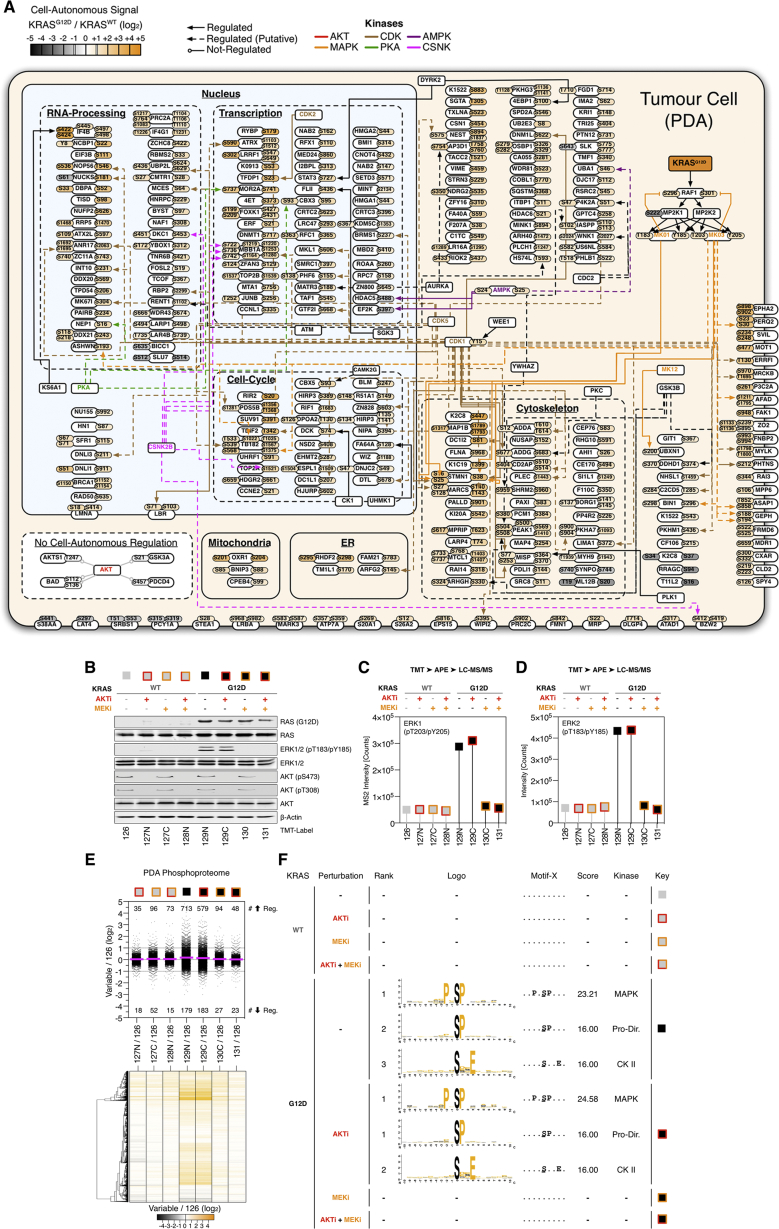
Cell-Autonomous KRAS^G12D^ Phosphoproteome, Related to Figure 2 (A) Cellular heat map of phosphoproteomic data described in Figure 2. Phosphosites in PDA tumor cells ± 1 log_2_, p < 0.01 (two-tailed t test) following KRAS^G12D^ induction (Uniprot annotation). Parent kinases are assigned as empirical (Uniprot) or putative (Scansite 3.0, ‘High-Stringency’, top 0.2% percentile). Cell-autonomous KRAS^G12D^ signaling is largely dictated by MAPK1/3 and CDK1. No cell-autonomous regulation of AKT substrates was observed. (B) PDA cells were cultured ± KRAS^G12D^ (1 μg/mL doxycycline) +/− AKTi (500 nM MK2206), +/− MEKi (500 nM PD 184352) or vehicle control for 12 hr. Immuno-blot analysis confirms expression of KRAS^G12D^, ERK1/2 phosphorylation, and MEKi / AKTi activity. Each condition was individually digested, TMT-labeled, pooled, enriched for phosphopeptides and analyzed by LC-MS/MS. (C) Raw product ion TMT intensities for pERK1 (pT203/pY205). (D) Raw product ion TMT intensities for pERK2 (pT183/pY185). (E) Differential phosphopeptide abundance across all variables (regulated = +/− 1 log_2_) as data-spread (bold line = replicate mean) and hierarchical clustered heatmap. (F) Motif-X analysis of upregulated (variable / 126 = log_2_ ≥ 1) phosphopeptides. Active ERK conditions (KRAS^G12D^, no MEK inhibitor) demonstrate enriched MAPK, Pro-Directed and CK II motifs. No regulated motifs were enriched from inactive ERK conditions (with MEK inhibitor).

**Figure S3:**
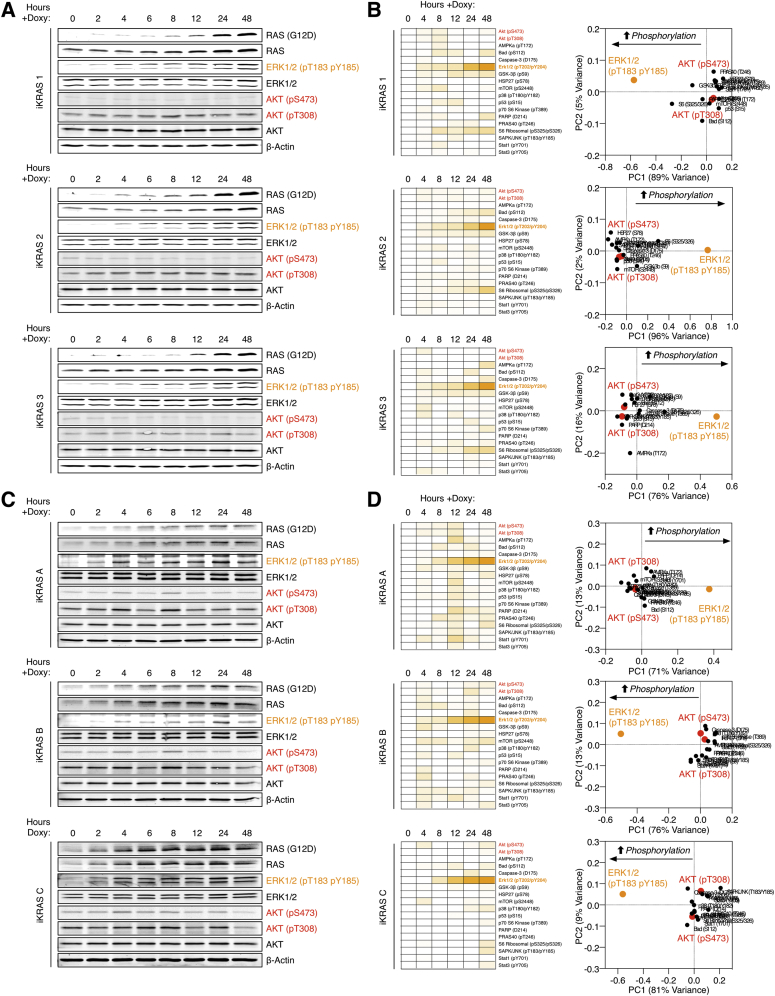
PDA KRAS^G12D^ Expression Regulates Cell-Autonomous ERK1/2, but Not AKT, Related to Figure 2 (A) iKRAS PDA cells (1, 2 and 3) were switched from KRAS^WT^ (0 hr) to KRAS^G12D^ (via doxycycline) across 48 hr. Phosphorylated ERK1/2 (pT183/pY185) and AKT (pS473; pT308) were assessed by Western blot. While KRAS^G12D^ expression closely correlates with phosphorylated ERK1/2 (pT183/pY185), AKT (pS473; pT308) is not regulated. (B) PDA cells were switched from KRAS^WT^ (0 hr) to KRAS^G12D^ (via doxycycline) across 48 hr. 18 intracellular signaling nodes were monitored using a reverse-phase antibody capture array. In agreement with Western blot analysis, induction of KRAS^G12D^ expression leads to upregulation of ERK1/2 (pT183/pY185), but does not regulate AKT (pS473; pT308) or AKT substrates. (C) Identical experiment to (A), but with PDA cells (A–C) described by Collins et al. (2012). In these distinct PDA cells, KRAS^G12D^ expression also correlates with phosphorylated ERK1/2 (pT183/pY185), whereas cell-autonomous epithelial KRAS^G12D^ does not regulate AKT (pS473; pT308). (D) Identical experiment to b) but with iKRAS cells from Collins et al., 2012. Again, KRAS^G12D^ upregulates ERK1/2 (pT183/pY185), but does not regulate AKT (pS473; pT308) or AKT substrates.

To provide a baseline of cell-autonomous oncogene-regulated signaling from which to compare stromal-dependent reciprocal signaling, we first determined the effect of KRAS^G12D^ expression on the PDA phosphoproteome (Figures 2A, 2B, and S2A). Despite being the primary oncogenic driver in PDA, KRAS^G12D^ only regulates 7% of the observed tumor cell phosphoproteome (+/−1 log_2_, p < 0.01) (Figure 2C; Data S1). KRAS^G12D^ expression induces canonical activation of ERK1/2 and increases phosphorylation of MAPK/CDK1/CKII-directed kinase motifs. However, while the PI3K-AKT axis is often presumed directly downstream of KRAS^G12D^ in PDA (Eser et al., 2014)—expression of KRAS^G12D^ does not activate AKT in a cell-autonomous manner (Figures 2D and S2). This observation is consistent across multiple PDA cell isolations from several independently developed genetic mouse models (Collins et al., 2012, Ying et al., 2012) (Figure S3). To further investigate the dependency of MEK and AKT activity in KRAS^G12D^ cell-autonomous signaling, KRAS^WT^ and KRAS^G12D^ PDA cells were perturbed with MEK (PD-184352) and/or AKT (MK-2206) inhibitors and analyzed by quantitative phosphoproteomics. This analysis confirmed MEK-ERK1/2, not AKT, controls the differential phosphoproteome of KRAS^G12D^ (Figure 2E; Data S1).

Collectively, these observations demonstrate cell-autonomous KRAS^G12D^ regulates a distinct section of the tumor cell phosphoproteome. Notably, KRAS^G12D^ induces MAPK/CDK/CK kinase motifs via MEK-ERK and does not regulate AKT.

### Activated Stromal Cells Extend Tumor Cell Signaling beyond Cell-Autonomous KRAS^G12D^

**Figure 3:**
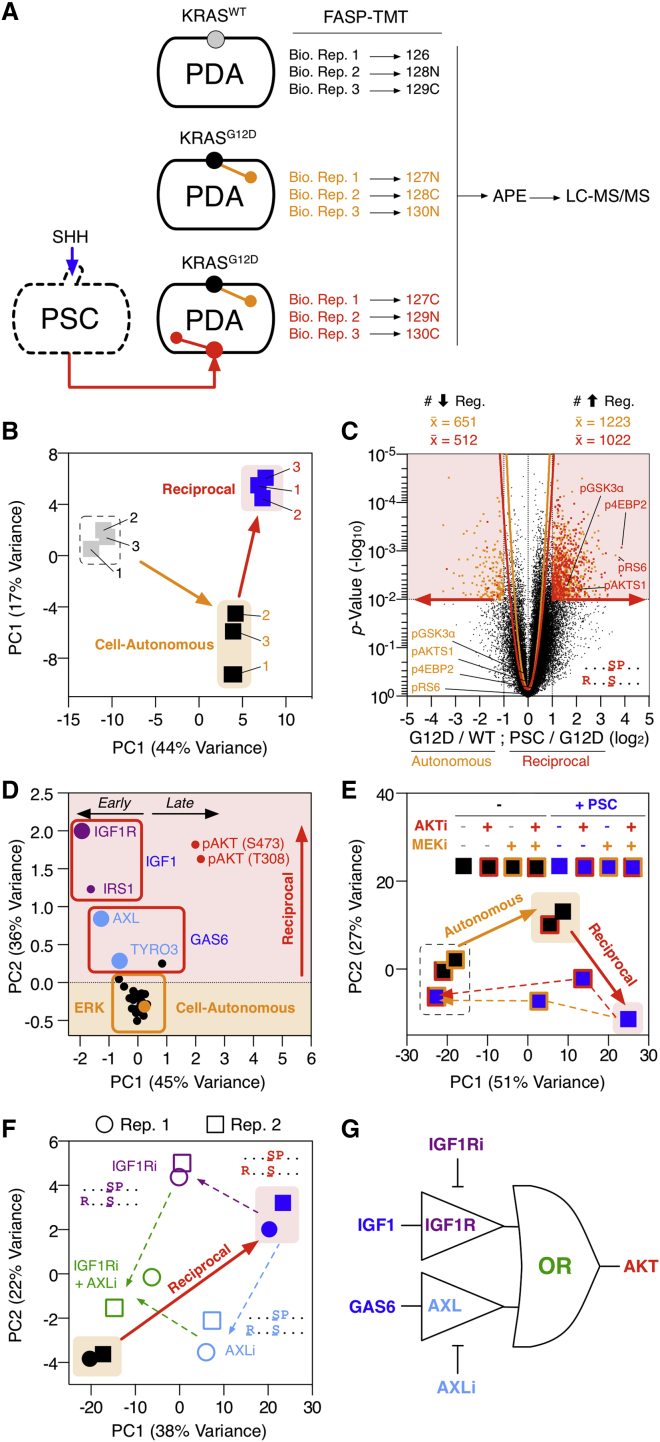
Activated Stromal Cells Regulate Tumor Cell Signaling beyond Cell-Autonomous KRAS^G12D^ (A) Multi-axis phosphoproteomics workflow allows concurrent comparison of different signaling inputs (n = 3). (B) PCA distribution of multi-axis phosphoproteomics. Conditioned medium from SHH-activated PSCs distinctly regulate the PDA phosphoproteome beyond cell-autonomous KRAS^G12D^ (n = 3). (C) Multi-axis double volcano phosphoproteome (both cell-autonomous (orange) and reciprocal (red) axis shown). Conditioned medium from activated PSCs regulate AKT substrates and AKT motifs in KRAS^G12D^ PDA cells. (D) Phospho-nodes regulated in PDA tumor cells treated with PSC conditioned media ± SHH across 30 min. Activated PSCs regulate PDA IGF1R/IRS-1, AXL/TYRO-3 (2.5 min), and AKT (>5 min) phosphorylation. (E) KRAS^G12D^ PDA phosphoproteome ± PSC+SHH conditioned media, +/− MEK and AKT inhibitors. Unlike cell-autonomous KRAS^G12D^, the reciprocal PDA phosphoproteome signaling state requires both MEK and AKT activity. (F) KRAS^G12D^ PDA phosphoproteome ± PSC+SHH conditioned media, +/− IGF1R and AXL inhibitors. Combined perturbation of IGF1R and AXL is required to partially restore the PDA cell-autonomous state. (G) PDA molecular logic model. See also Figure S4 and Data S1.

Given that KRAS^G12D^ non-cell-autonomously regulates growth factor production from PSCs (e.g., IGF1 and GAS6), we hypothesized that KRAS^G12D^-activated PSCs initiate a reciprocal signaling axis back in the tumor cells. However, given that tumor cells already undergo phosphoproteomic deregulation by KRAS^G12D^, it was unclear whether additional reciprocal signals from PSCs can further regulate the tumor cell phosphoproteome. To investigate this, the phosphoproteome of KRAS^WT^ and KRAS^G12D^ PDA cells were directly compared to PDA cells treated with conditioned media from SHH-activated PSCs (Figure 3A).

**Figure S4:**
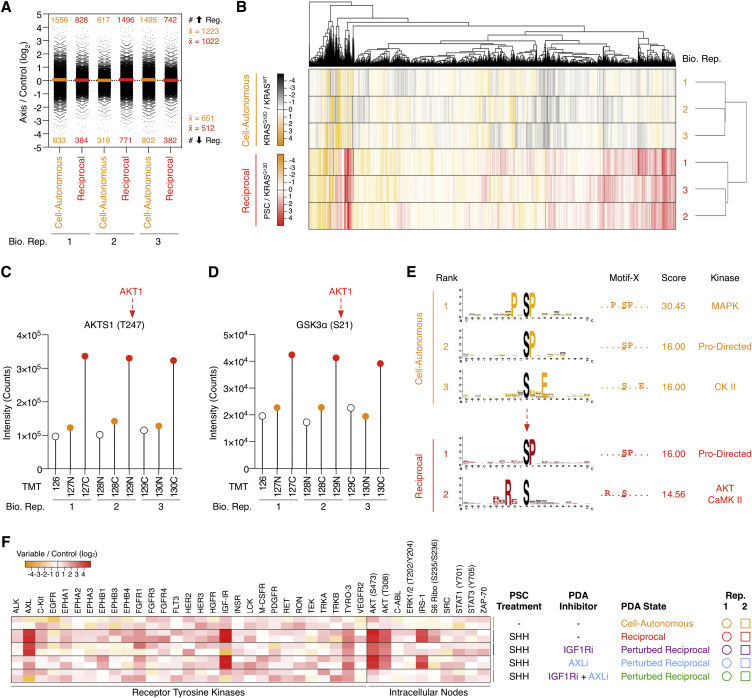
Multi-axis Phosphoproteomics, Related to Figure 3 (A) Differential PDA phosphopeptide distributions across all biological replicates as data spread (bold line = replicate mean) from the experiment described in Figure 3A. (B) Hierarchal clustering of multi-axis phosphoproteomic biological replicates group each signaling axis. (C) Raw TMT product ion intensity spectra of the AKT substrate AKTS1 (pT247). (D) Raw TMT product ion intensity spectra of the AKT substrate GSK3α (pS21). (E) Motif-X analysis of upregulated (log_2_ ≥ 1) phosphopeptides. Cell-autonomous KRAS^G12D^ regulates MAPK, CDK and CK2 motifs. Reciprocal signaling introduces AKT/CaMK II motif regulation. (F) PDA (KRAS^G12D^) receptor tyrosine kinase (RTK) and intracellular node phosphorylation following treatment with PSC conditioned medium ± SHH for 2.5 min. Combined PDA pre-treatment with IGF1R inhibitor (250 nM Picropodophyllin (PPP)) and AXL inhibitor (500 nM R428) is required to block early AKT phosphorylation.

Despite the considerable regulation of cell-autonomous signaling by KRAS^G12D^, PDA cells are further modulated by signals from SHH-activated PSCs (Figure 3B). In fact, PSC-signaling regulates (+/−1 log_2_, p < 0.01) comparable numbers of PDA tumor cell phosphosites (6.7% phosphoproteome) when compared to KRAS^G12D^ alone (7.2% phosphoproteome) (Figures 3C, S4A, and S4B; Data S1). This implies stromal cells can substantially alter tumor cell signaling beyond cell-autonomous KRAS^G12D^. Notably, while PDA KRAS^G12D^ expression does not activate AKT in a cell-autonomous manner (Figures 2 and S3), tumor cell AKT substrate phosphosites (e.g., AKTS1 [pT247] and GSK3α [pS21]) are exclusively regulated by stromal PSCs (Figures S4C–S4E).

Targeted temporal analysis revealed SHH-activated PSCs induce rapid phosphorylation of IGF1R (receptor for IGF-1), AXL/TYRO3 (receptor for GAS6), and downstream IRS-1 and AKT (pT308/pS473) in KRAS^G12D^ PDA cells (Figure 3D). Tumor cells treated with conditioned media from control or SHH-activated PSCs and perturbed with either MEK and/or AKT inhibitors further confirmed PSCs drive a differential phosphoproteome in PDA cells. However, unlike cell-autonomous KRAS^G12D^, stromal-driven signaling depends on both active MEK and AKT (Figure 3E; Data S1).

As IGF1 and GAS6 are secreted by activated PSCs, we investigated the dependency of IGF1R and AXL activity on the PSC-induced tumor cell phosphoproteome. Combined IGF1R and AXL inhibitors are required to block the PSC-induced tumor cell phosphoproteome—suggesting a Boolean “OR” axis between PSC IGF1/GAS6 and PDA pAKT (Figures 3F, 3G, and S4F; Data S1).

Collectively, these results reveal activated stromal cells can return a differential signal to tumor cells via an IGF1R/AXL-AKT axis. The stromal-driven tumor cell phosphoproteome is distinct from the KRAS^G12D^ regulated cell-autonomous phosphoproteome and responds differently to pharmacological perturbation.

### KRAS^G12D^ Regulates Tumor Cell Signaling via a Reciprocal Signaling Axis

Our data suggests that oncogenic KRAS in tumor cells establishes a reciprocal signaling axis between stromal cells and tumor cells. Herein, we define an oncogenic reciprocal signaling axis as an oncogenic cue that signals via an adjacent heterotypic cell to produce a distinct response in the oncogene-expressing cell. For this heterocellular variation on the “cue-signal-response” systems biology paradigm (Janes et al., 2004, Janes et al., 2005, Miller-Jensen et al., 2007) to be valid, we hypothesized that oncogenic reciprocal signaling requires three essential features: (1) an oncogenic cue (e.g., KRAS^G12D^), (2) a cue-driven non-cell-autonomous signal (e.g., KRAS^G12D^-induced SHH), and (3) a heterotypic cell capable of transducing the signal response back to the instigating oncogenic cell (e.g., PSC). To test this multi-node reciprocal signaling hypothesis, we systematically perturbed each reciprocal feature in a native heterocellular tumor-stroma context.

**Figure 4:**
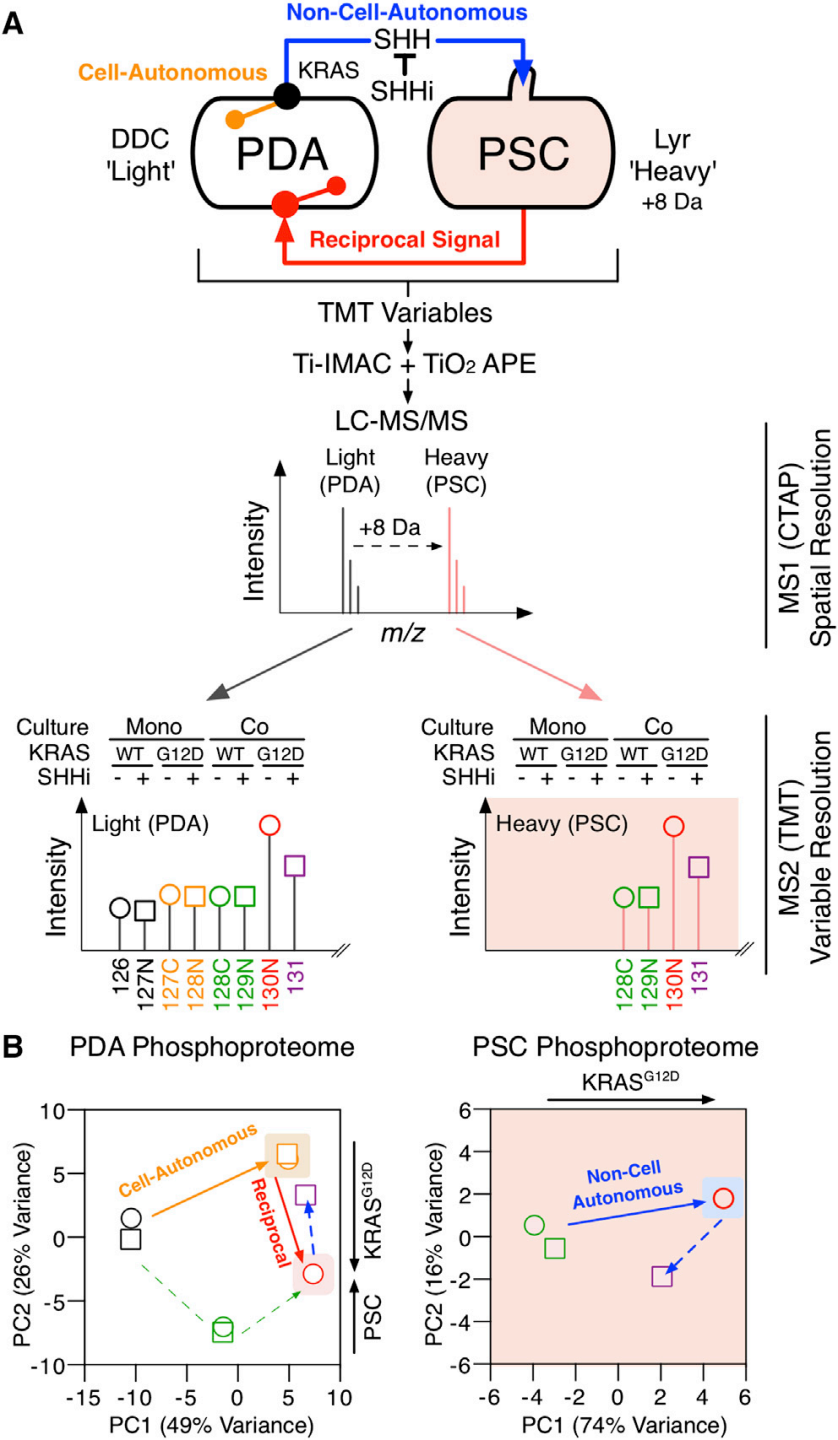
KRAS^G12D^ Heterocellular Reciprocal Signaling (A) Heterocellular multivariate phosphoproteomic workflow. CTAP “Light” PDA+DDC^*M.Tub*-KDEL^ cells ± KRAS^G12D^, +/− SHHi, and +/− “Heavy” PSC+Lyr^M37-KDEL^. Each variable was TMT-labeled and enriched for phosphopeptides (by APE). CTAP labeling provides cell-specific data (MS1 scan) and TMT labeling provides variable-specific data (MS2 scan). (B) Concurrent measurement of cell-autonomous, non-cell-autonomous, and reciprocal phosphoproteomes in a heterocellular environment. Oncogenic reciprocal signaling requires a mutational cue, a trans-cellular signal, and a heterocellular context. See also Figure S5 and Data S1.

To measure multivariate signaling in a heterocellular system, concurrent cell-specific and variable-specific phosphoproteomic data are required. We have previously shown that stable isotopic proteome labeling (Ong et al., 2002) can resolve between discrete cell types in direct culture of heterotypic cells (Jorgensen et al., 2009) and recently introduced cell type-specific labeling with amino acid precursors (CTAP) (Gauthier et al., 2013) Lyr^M37-KDEL^ and DDC^*M.tub*-KDEL^ enzymes for cell-specific isotopic labeling (Tape et al., 2014a). To this end, we combined CTAP labeling (spatial resolution) with isobaric tandem mass tag (TMT) phosphoproteomics (variable resolution) (Tape et al., 2014b, Thompson et al., 2003) to enable heterocellular multivariate phosphoproteomic analysis of each reciprocal signaling component (Figure 4A). This technique allows simultaneous observation of cell-autonomous, non-cell-autonomous, and reciprocal oncogenic phosphoproteomes at cell-specific resolution.

**Figure S5:**
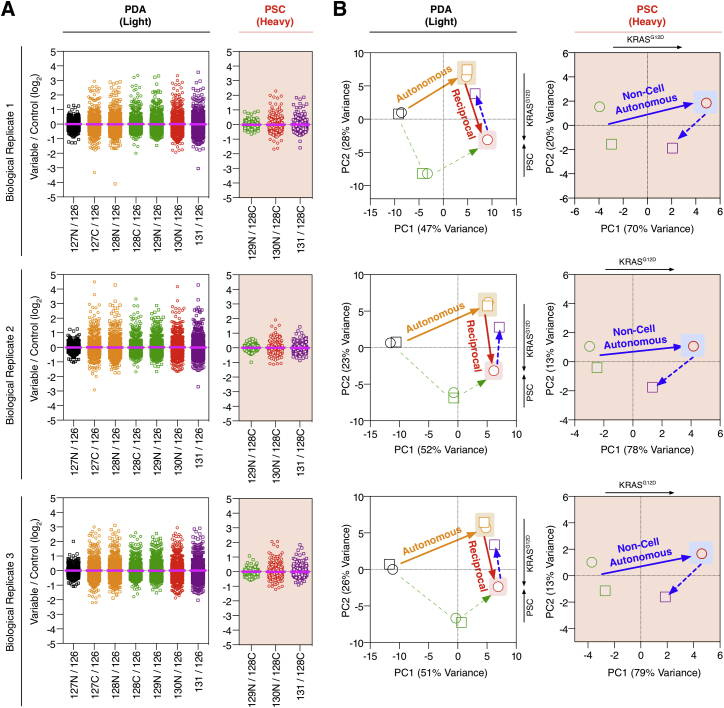
Biological Replicates of Heterocellular Multivariate Phosphoproteomics, Related to Figure 4 (A) Cell-specific differential phosphopeptide abundance from CTAP ‘Light’ PDA+DDC^*M.Tub*-KDEL^ and ‘Heavy’ (K +8 Da) PSC+Lyr^M37-KDEL^ cells across all variables and replicates (bold line = variable mean) (from Figure 4). (B) Differential cell-specific phosphoproteomic PCA states for each replicate. KRAS^G12D^, active SHH and PSCs (reciprocal signaling axis) achieve a distinct phosphoproteomic state. (Cell-autonomous axis, orange; non-cell-autonomous, blue; reciprocal axis, red; non-oncogene driven stromal, green.)

Cell-specific phosphoproteomes were interrogated in PDA cells expressing either KRAS^WT^ or KRAS^G12D^, either in homo- or heteroculture with isotopically “heavy”-labeled PSCs, and treated with either SHH inhibitor or vehicle. We monitored 3,695 lysine-containing (8,566 total) phosphopeptides across eight conditions, two heterotypic cell types, and three biological replicates with cell-specific resolution (Figures 4B and S5; Data S1). As expected, expression of KRAS^G12D^ in tumor cells alone regulates (+/−1 log_2_) 7.2% of the identified cell-autonomous phosphoproteome. In parallel, tumor cell KRAS^G12D^ non-cell-autonomously regulates 4.7% of the PSC phosphoproteome. Moreover, when KRAS^G12D^ is allowed to communicate with PSCs via SHH, a reciprocal axis is completed and the differentially regulated tumor cell phosphoproteome almost doubles to 13.8%. Importantly, perturbation by a SHH blocking antibody decreases the phosphoproteomic regulation on PSCs back down to 1.2% and PDA phosphoproteome to 8.1% (close to cell-autonomous at 7.2%).

Heterocellular multivariate phosphoproteomics demonstrates how tumor cell oncogenes exploit the differential signaling capacity of stromal cells to achieve a unique signaling state in the inceptive tumor cell. KRAS^G12D^ reciprocal signaling engages additional phospho-nodes to cell-autonomous KRAS^G12D^ alone, allowing KRAS^G12D^ to extend the oncogenic signaling capacity in the inceptive tumor cells. Crucially, these observations are the product of native tumor-stroma signaling and are independent of exogenous stimulation.

### KRAS^G12D^-Driven Reciprocal Signaling Regulates the Tumor Cell Phosphoproteome and Total Proteome

**Figure 5:**
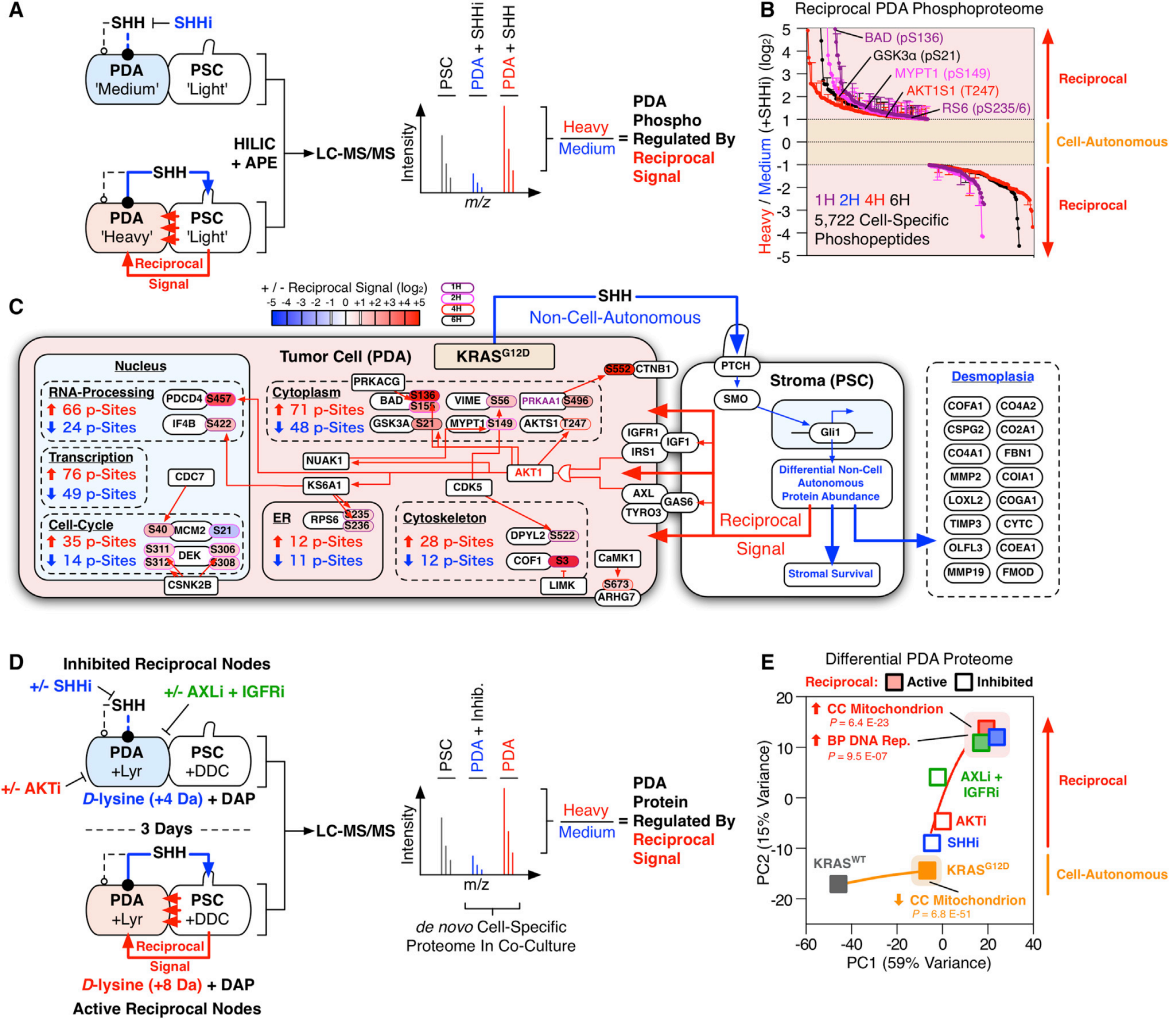
Reciprocal Signaling Regulates the Tumor Cell Phosphoproteome and Total Proteome (A) Comprehensive reciprocal signaling phosphoproteomic workflow. PDA cells were SILAC-labeled “Heavy” or “Medium” and co-cultured with “Light” PSCs pre-activated ± SHH respectively. Heterocellular proteomes were co-fractionated by HILIC and automatically enriched for phosphopeptides (by APE). When analyzed by LC-MS/MS, “Heavy”/”Medium” ratios report differential PDA phosphoproteome regulation in a heterocellular context. (B) Reciprocal signaling differential regulates the PDA phosphoproteome (including AKT substrates). (C) Heterocellular oncogenic signaling summary. AKT signaling, RNA-processing, and transcriptional regulation are regulated in PDA tumor cells by reciprocal signaling. (D) Isotopically CTAP-labeled PDA+Lyr^M37-KDEL^ cells and PSC+DDC^*M.tub*-KDEL^ cells were continuously co-cultured ±SHHi, AKTi, or IGF1Ri + AXLi reciprocal node inhibitors. When analyzed by LC-MS/MS, “Heavy”/“Medium” ratios report differential PDA proteome in a heterocellular context. (E) Reciprocal signaling produces a differential proteomic state (including mitochondrial and DNA replication proteins) in PDA cells. Second order polynomial regression. See also Figure S6 and Data S1.

**Figure S6:**
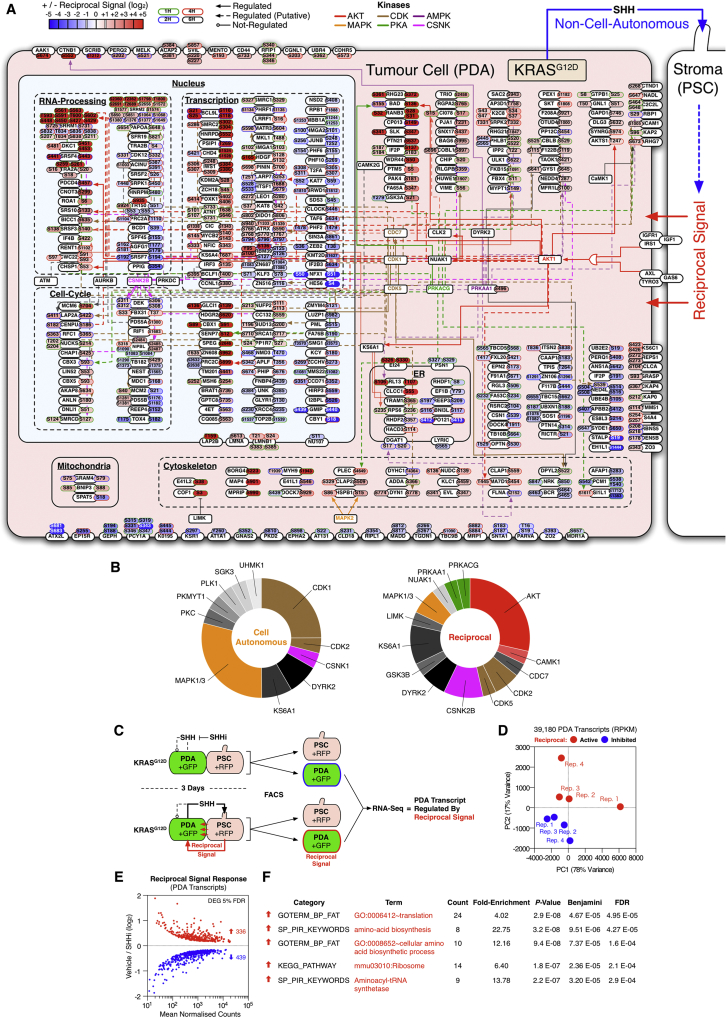
PDA Reciprocal Phosphoproteome and Transcriptome, Related to Figure 5 (A) Cellular heat map of phosphoproteomic data described in Figure 5. Phosphosites in PDA tumor cells ± 1 log_2_, following reciprocal signal induction. Parent kinases are assigned as empirical (Uniprot) or putative (Scansite 3.0 ‘High-Stringency’, top 0.2% percentile). Reciprocal signaling upregulates AKT substrates and modifies proteins involved in RNA-processing and transcriptional regulation. (B) Uniprot parent kinase annotations of upregulated (≥1 log_2_) phosphosites by cell-autonomous (n = 24) and reciprocal (n = 28) signaling axis. Cell-autonomous KRAS^G12D^ signaling is dominated by CDK1 and MAPK1/3 activity. No AKT substrates are regulated by cell-autonomous KRAS^G12D^. Conversely, reciprocal KRAS^G12D^ signaling regulates multiple AKT substrates and does not modulate any CDK1 substrates. (C) RNA-seq workflow. PDA+GFP cells were co-cultured with PSC+RFP cells ± SHHi for 3 days. PDA cells were resolved by FACS and subjected to RNA-seq analysis (n = 4). (D) PCA distribution of reads per killable per million mapped reads (RPKM) values. (E) Differentially expressed genes (DEG) at 5% FDR. (F) DAVID functional GO-enrichment analysis of upregulated DEGs (p < E-06). Reciprocal signaling upregulates transcripts associated with protein translation and amino acid biosynthesis in PDA cells.

Comprehensive phosphoproteomic quantification of reciprocally engaged PDA cells (Figures 5A–5C and S6A; Data S1) revealed upregulation of several AKT substrates (e.g., BAD [pS136], PDCD4 [pS457], CHSP1 [pS53], AKTS1 [T247], and GSK-3α [pS21]). Interestingly, cell-autonomous targets of KRAS^G12D^ (e.g., RAF1 [pS621] and ERK1/2 [pT183/pY185; pT203/pY205]) were not regulated by reciprocal signaling—further implying reciprocal KRAS^G12D^ supplements cell-autonomous KRAS^G12D^ by engaging additional tumor cell kinases (Figure S6B).

Reciprocal signaling also activates several translational mediators (e.g., RPS6 [pS235/pS236], PDCD4 [pS457], and EIF4B [pS422]). Concordantly, RNA sequencing (RNA-seq) analysis of PDA cells revealed reciprocal signaling upregulates RNA associated with translational control (Figures S6C–S6F), further suggesting a de novo control of PDA protein abundance. To validate whether the SHH-driven reciprocal signaling axis regulates de novo tumor cell protein turnover, PSC+DDC^*M.tub*-KDEL^ and PDA+Lyr^M37-KDEL^ CTAP cells were differentially isotopically labeled, treated with a SHH inhibitor or vehicle, and cell-specific proteomes were quantified in heteroculture (Figure 5D). This experimental format permitted cell-specific quantification of changes to the KRAS^G12D^ tumor cell proteome following inhibition of the PSC targeting signal (SHH). Parallel perturbations with AKT and IGF1R/AXL inhibitors provided additional insight into the role of each reciprocal node.

Cell-specific proteomics confirmed KRAS^G12D^ reciprocally regulates the PDA proteome and is dependent on active SHH, IGF1R/AXL, and AKT signaling (Figure 5E; Data S1). As with the PDA phosphoproteome, reciprocal signaling regulates the PDA proteome differently to cell-autonomous KRAS^G12D^. For example, while cell-autonomous KRAS^G12D^ rapidly depletes distinct mitochondrial components from PDA cells (Data S1) (Viale et al., 2014), reciprocally engaged KRAS^G12D^ restores mitochondrial proteins in an SHH-, IGF1R/AXL-, and AKT-dependent manner. Moreover, PDA proteins involved with DNA replication are also upregulated under reciprocal conditions. These results demonstrate reciprocal signaling uniquely regulates both the tumor cell phosphoproteome and global proteome when compared to cell-autonomous signaling. Reciprocal signaling states are unique to a heterocellular environment and are not observed in tumor cells alone.

### KRAS^G12D^-Driven Reciprocal Signaling Regulates Tumor Cell Phenotypes

**Figure 6:**
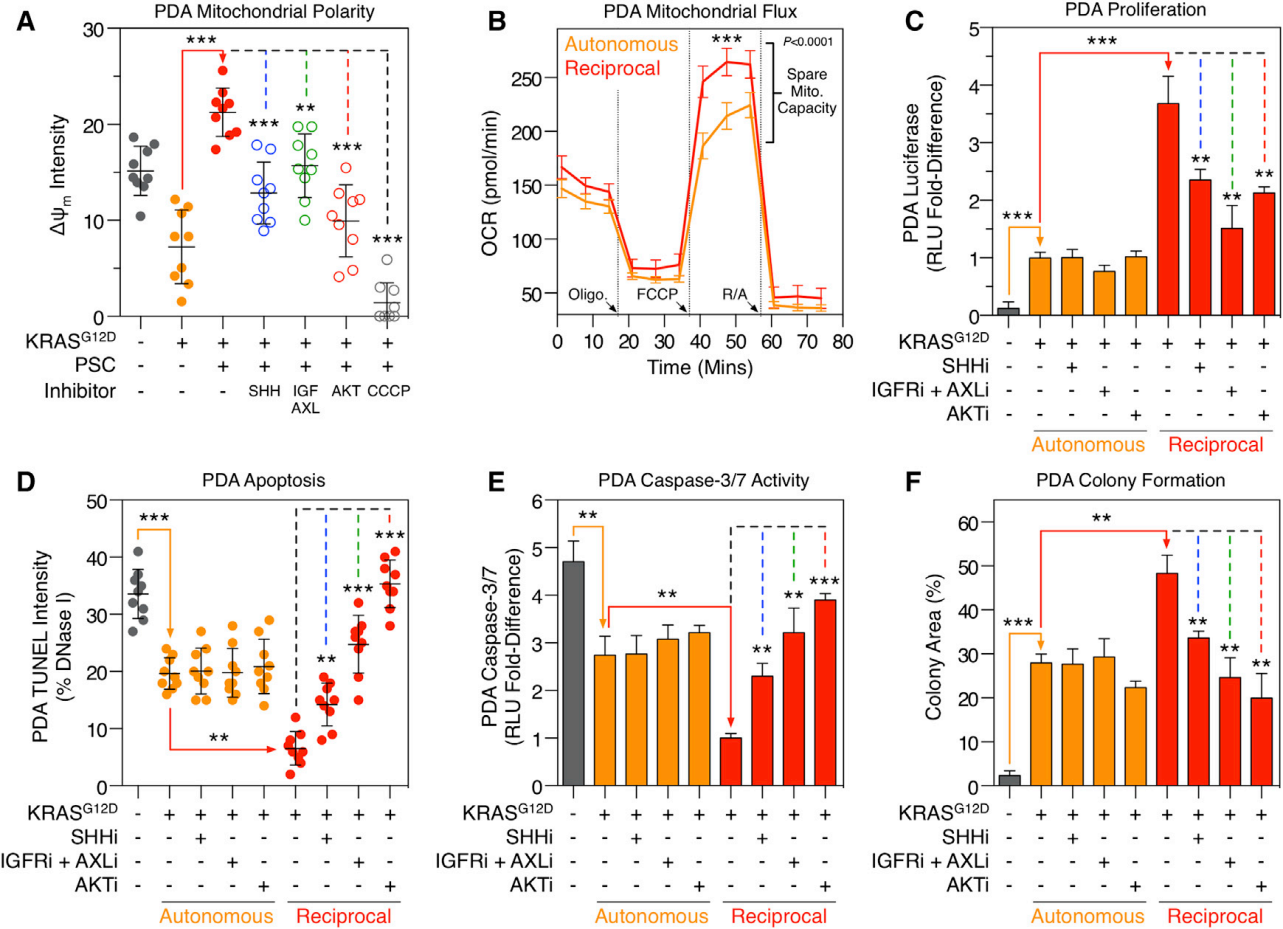
Reciprocal Signaling Regulates Tumor Cell Phenotypes (A) High-content live-cell TMRE analysis of PDA mitochondrial polarity. As predicted by heterocellular proteomics, reciprocal signaling restores mitochondrial polarity via SHH, IGF1R/AXL, and AKT (Δψ_m_) (n = 9). ^∗^p < 0.05, ^∗∗^p < 0.01, ^∗∗∗^p < 0.001. (B) PDA mitochondrial flux analysis. As predicted by heterocellular proteomics, reciprocal signaling increases spare mitochondrial capacity when compared to cell-autonomous KRAS^G12D^ alone (two-way ANOVA). OCR, oxygen consumption rate. ^∗^p < 0.05, ^∗∗^p < 0.01, ^∗∗∗^p < 0.001. (C) Cell-autonomous and reciprocal proliferation of luciferase-labeled tumor cells. Reciprocal KRAS^G12D^ (heterocellular, red) increases PDA proliferation relative to cell-autonomous KRAS^G12D^ (homocellular, orange). Inhibitors of reciprocal nodes only perturb heterocellular tumor cells (n = 3). ^∗^p < 0.05, ^∗∗^p < 0.01, ^∗∗∗^p < 0.001. (D) High-content TUNEL imaging of PDA apoptosis. Reciprocal signaling protects tumor cells from apoptosis beyond cell-autonomous KRAS^G12D^. Inhibiting IGF1R/AXL or AKT increases apoptosis when reciprocal signaling is active (n = 9). ^∗^p < 0.05, ^∗∗^p < 0.01, ^∗∗∗^p < 0.001. (E) Caspase 3/7 activity in (D) (n = 3). ^∗^p < 0.05, ^∗∗^p < 0.01, ^∗∗∗^p < 0.001. (F) Semi-solid PDA colony formation. Reciprocal signals increase colony formation (via SHH, IGF1R/AXL, and AKT) relative to cell-autonomous KRAS^G12D^ alone (n = 3). ^∗^p < 0.05, ^∗∗^p < 0.01, ^∗∗∗^p < 0.001. See also Figure S7.

**Figure S7:**
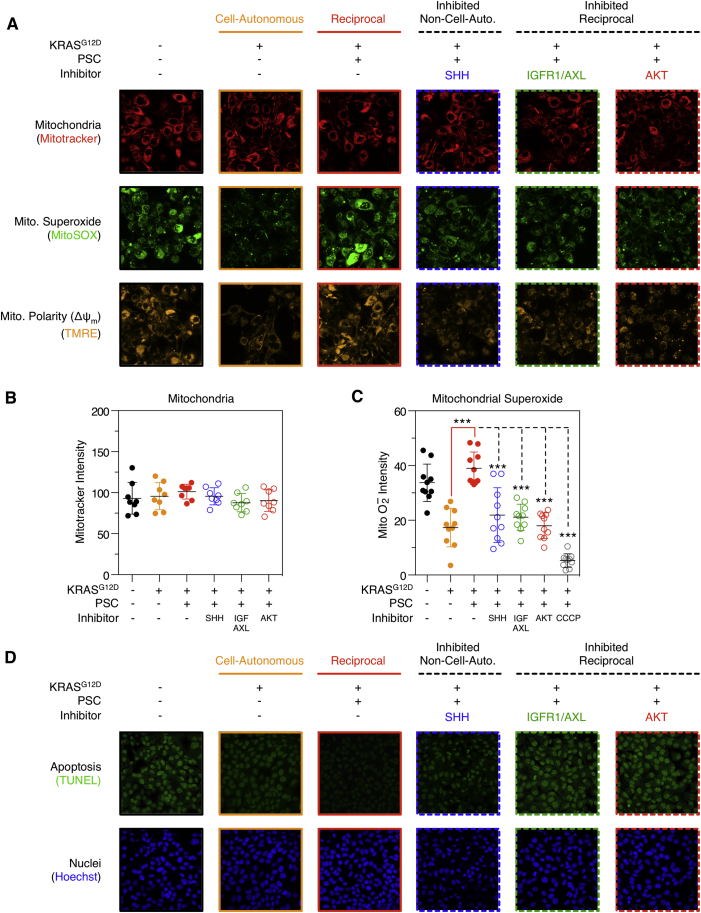
KRAS^G12D^ Cell-Autonomous and Reciprocal Regulation of PDA Mitochondria, Related to Figure 6 (A) PDA tumor cells stained for total mitochondria (MitoTracker), mitochondrial superoxide (MitSOX) and mitochondrial polarity (Δψ_m_) (TMRE). While cell-autonomous and reciprocal KRAS^G12D^ does not alter total mitochondria staining, reciprocal signaling upregulates mitochondrial superoxide and mitochondrial polarity. (B and C) High-content imaging quantification of mitochondrial intensity and superoxide (two-tailed t test: ^∗^ = p < 0.05, ^∗∗^ = p < 0.01, ^∗∗∗^ = p < 0.001) (all error bars = SD, n = 10). (D) High-content imaging of TUNEL and Hoechst stained PDA cells.

Reciprocal signaling regulates proteins and phospho-sites known to control several important biological processes. For example, while cell-autonomous and reciprocal KRAS^G12D^ signaling both regulate mitochondrial proteins, many of these are asymmetrically regulated. As a result, we hypothesized PDA mitochondrial activity would be differentially regulated by cell-autonomous and reciprocal KRAS^G12D^. Concordantly, cell-autonomous KRAS^G12D^ decreases PDA mitochondria polarization (Δψ_m_) and mitochondrial superoxide production, whereas reciprocal signaling increases these processes (via SHH, IGF1R/AXL, and AKT) (Figures 6A and S7). Furthermore, reciprocal signaling increases spare mitochondrial respiratory capacity in tumor cells (Figure 6B). These results demonstrate KRAS^G12D^ can differentially regulate mitochondrial performance via heterocellular communication.

Reciprocal signaling also regulates proteins known to control cell proliferation and survival. In agreement, cell-specific analysis of PDA proliferation in homo and heterocellular cultures revealed increased tumor cell proliferation under heterocellular conditions (via SHH, IGF1R/AXL, and AKT activity) (Figure 6C). Upregulation of AKT substrates (e.g., inhibition of BAD [pS136]) also suggested reciprocal signaling might protect tumor cells from apoptosis. Concordantly, TUNEL and caspase 3/7 profiling revealed activated PSCs protect tumor cells from apoptosis and sensitize tumor cells to reciprocal node inhibitors (IGF1R/AXL and AKT) (Figures 6D–6E).

Increased mitochondrial performance, proliferative capacity, and resistance to apoptosis collectively implied reciprocal signaling supports tumor cell phenotypes beyond cell-autonomous KRAS^G12D^. In accordance, reciprocal signaling increases semi-solid colony growth relative to cell-autonomous KRAS^G12D^ alone (Figure 6F). Reciprocal colony growth is dependent on SHH activation of PSCs and IGF1R/AXL-AKT activity in tumor cells. Collectively, these results demonstrate the unique signals produced by reciprocal KRAS^G12D^ control distinct metabolic, proliferative, anti-apoptotic, and anchorage-independent growth phenotypes in tumor cells.

## Discussion

**Figure 7:**
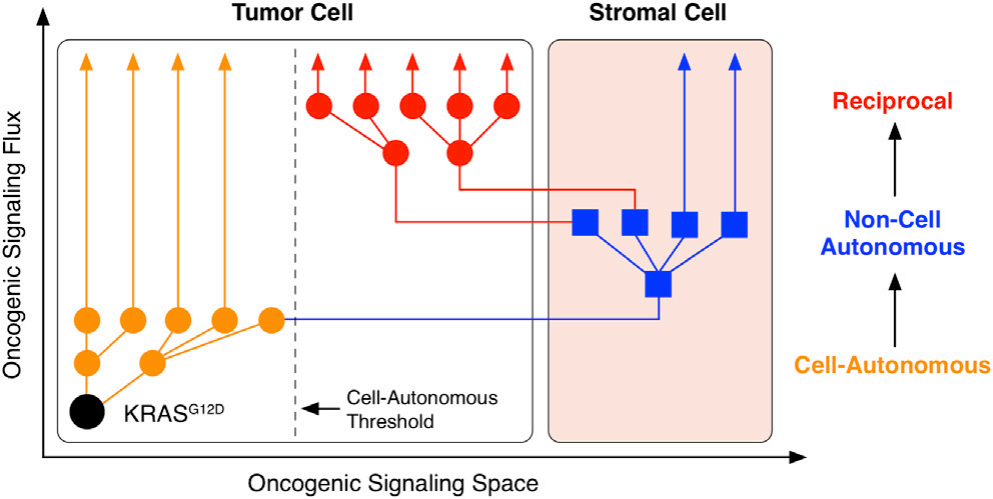
Heterocellular Oncogenic Signaling In a homocellular context, tumor cell oncogenic signaling operates within distinct cell-autonomous phospho-networks. As heterotypic cell types can transduce different signals, a heterocellular system provides increased oncogenic signaling space over a homocellular system. Tumor cells can use heterocellularity to bypass the cell-autonomous threshold via non-cell-autonomous signaling. Activated stromal cells can then return unique reciprocal signals to the initiating oncogenic tumor cell. Reciprocal signaling subsequently allows oncogenes to adopt a tumor cell oncogenic signaling space beyond cell-autonomous signaling alone.

Whether oncogenes regulate tumor cell signaling via stromal cells is a fundamental question in tumor biology. Using heterocellular multivariate phosphoproteomics, we demonstrate how oncogenic KRAS signals through local non-tumor cells to achieve a differential reciprocal signaling state in the inceptive tumor cells. In PDA, this reciprocal axis supplements oncogenic cell-autonomous signaling to control protein abundance, transcription, mitochondrial activity, proliferation, apoptosis, and colony formation. Reciprocal signaling is the exclusive product of heterocellularity and cannot be achieved by tumor cells alone. These observations imply oncogenes expand their capacity to deregulate cellular signaling via stromal heterocellularity (Figure 7).

Despite the well-established heterocellularity of cancer, our understanding of oncogenic signaling within tumor cells has largely excluded non-tumor cells. We observe that stromal cells approximately double the number of tumor cell signaling nodes regulated by oncogenic KRAS, suggesting both cell-autonomous (internal) and reciprocal (external) stimuli should be considered when defining aberrant oncogenic signaling states. For example, although KRAS is thought to cell-autonomously regulate AKT in PDA (Eser et al., 2014), we show that KRAS^G12D^ activates AKT, not cell-autonomously, but reciprocally. As PI3K signaling is essential for PDA formation in vivo (Baer et al., 2014, Eser et al., 2013, Wu et al., 2014) reciprocal signaling may control oncogene-dependent tumorigenesis. Our findings suggest future genetic studies should consider the heterocellular signaling consequences of oncogene/tumor-suppressor deregulation.

The observation that many oncogene-dependent tumor cell signaling nodes require reciprocal activation has important implications for identifying pharmacological inhibitors of oncogene signaling. For example, if PDA tumor cells were screened alone, one would expect MEK, MAPK, and CDK inhibitors to perturb KRAS^G12D^ signaling. However, when screened in conjunction with heterotypic stromal cells, our study additionally identified SHH, AKT, and IGF1R/AXL inhibitors as KRAS^G12D^-dependent targets in tumor cells. Inhibitors of signaling specific to reciprocally engaged tumor cells, such as or AKT or IGF1R/AXL, block heterocellular phenotypes (e.g., protein expression, proliferation, mitochondrial performance, and anti-apoptosis), but have little effect on KRAS^G12D^ tumor cells alone. An appreciation of reciprocal nodes increases our molecular understanding of drug targets downstream of oncogenic drivers and highlights focal points where reciprocal signals converge (e.g., AKT). These trans-cellular observations reinforce the importance of understanding cancer as a heterocellular disease.

Previous work in PDA tumor cells under homocellular conditions demonstrated cell-autonomous KRAS^G12D^ shifts metabolism toward the non-oxidative pentose phosphate pathway (Ying et al., 2012), whereas KRAS^G12D^-ablated cells depend on mitochondrial activity (Viale et al., 2014). Here, we show that heterocellular reciprocal signaling can restore the expression of mitochondrial proteins and subsequently re-establish both mitochondrial polarity and superoxide levels. This suggests KRAS^G12D^ regulates non-oxidative flux through cell-autonomous signaling and mitochondrial oxidative phosphorylation through reciprocal signaling. These results provide a unique example of context-dependent metabolic control by oncogenes and reinforce the emerging role of tumor-stroma communication in regulating cancer metabolism (Ghesquière et al., 2014).

In PDA, the stroma has dichotomous pro-tumor (Kraman et al., 2010, Sherman et al., 2014) and anti-tumor (Lee et al., 2014, Rhim et al., 2014) properties. It is becoming increasingly evident that non-cell-autonomously activated stromal cells vary within a tumor and can influence tumors in a non-obvious manner. For example, while vitamin D receptor normalization of stromal fibroblasts improves PDA therapeutic response (Sherman et al., 2014), total stromal ablation increases malignant behavior (Lee et al., 2014, Rhim et al., 2014). Thus, while stromal purging is unlikely to provide therapeutic benefit in PDA, “stromal reprogramming” toward an anti-tumor stroma is now desirable (Brock et al., 2015). Although we describe a largely pro-tumor reciprocal axis, both pro- and anti-tumor stromal phenotypes likely transduce across reciprocal signaling networks. Our work suggests future efforts to therapeutically reprogram the PDA stroma toward anti-tumor phenotypes will require an understanding of reciprocal signaling. In describing the first oncogenic reciprocal axis, this study provides a foundation to measure the cell-cell communication required for anti-tumor stromal reprogramming.

We demonstrate heterocellular multivariate phosphoproteomics can be used to observe reciprocal signaling in vitro. Unfortunately, cell-specific isotopic phosphoproteomics is not currently possible in vivo. To delineate reciprocal signaling in vivo, experimental systems must support manipulation of multiple cell-specific variables and provide cell-specific signaling readouts. Simple pharmacological perturbation of reciprocal nodes (e.g., IGF1R, AXL, AKT, etc.) in existing PDA GEMMs will in principle affect all cell types (e.g., tumor cells, PSCs, immune cells) and cannot provide axis-specific information in vivo. Future in vivo studies of reciprocal signaling will require parallel inducible genetic manipulation (e.g., oncogene activation in cancer cell and/or inhibition of reciprocal node in stromal cell), combined with cell-specific signaling data (e.g., using epithelial tissue mass-cytometry) (Simmons et al., 2015).

We describe KRAS^G12D^ reciprocal signaling between PDA tumor cells and PSCs. However, it is likely oncogenic reciprocal signaling occurs across multiple different cell types in the tumor microenvironment. For example, in PDA, FAP^+^ stromal fibroblasts secrete SDF1 that binds tumor cells to suppress T cells (Feig et al., 2013). Our model predicts oncogene signaling expands across several cell types in the tumor microenvironment—including immune cells. Moreover, as oncogenes non-cell-autonomously regulate the stroma in many other tumor types (Croce, 2008), our model predicts oncogenic reciprocal signaling to be a broad phenomenon across all heterocellular cancers. The presented heterocellular multivariate phosphoproteomic workflow now enables future characterization of oncogenic reciprocal signaling in alternative cancer types.

As differentiated cells process signals in unique ways, heterocellularity provides increased signal processing space over homocellularity. We provide evidence that KRAS^G12D^ exploits heterocellularity via reciprocal signaling to expand tumor cell signaling space beyond cell-autonomous pathways. Given the frequent heterocellularity of solid tumors, we suspect reciprocal signaling to be a common—albeit under-studied—axis in oncogene-dependent signal transduction.

## Experimental Procedures

### KRAS^G12D^-Induced Soluble Signaling Molecules

KRAS^WT^ PDA cells (1 × 10^6^) were plated in a 6-well dish and cultured in DMEM + 0.5% FBS ± 1 μg/ml doxycycline for 72 hr. Conditioned media was analyzed for relative changes in KRAS^G12D^-driven cytokines and growth factors using the RayBio Mouse Cytokine Antibody Array G2000 (RayBiotech AAM-CYT-G2000-8) (144 proteins quantified in duplicate per sample). SHH-N expression after 24 hr was further validated by sandwich ELISA (R&D Systems DY461).

### KRAS^G12D^ Cell-Autonomous Signaling

For comprehensive phosphoproteomic quantification of KRAS^G12D^-dependent cell-autonomous signaling, 1 × 10^6^ KRAS^WT^ PDA cells were plated in a 6-well dish (DMEM + 0.5% FBS) and cultured ± 1 μg/ml doxycycline for 24 hr (biological replicates n = 5). Cells were lysed in 6 M urea, 10 mM NaPPi, 20 mM HEPES, pH 8.0, sonicated, centrifuged to clear cell debris, and protein concentration was determined by BCA (Pierce 23225). One hundred micrograms of each condition were individually digested by FASP (Wiśniewski et al., 2009), amine-TMT-10-plex-labeled (Pierce 90111) on membrane (iFASP) (McDowell et al., 2013), eluted, pooled, lyophilized, and subjected to automated phosphopeptide enrichment (APE) (Tape et al., 2014b). Phosphopeptides were desalted using OLIGO R3 resin (Life Technologies 1-1339-03) and lyophilized prior to liquid chromatography-tandem mass spectrometry (LC-MS/MS) analysis (see the Supplemental Experimental Procedures).

### Automated Phosphopeptide Enrichment

For TMT-labeled samples, phosphopeptides were enriched from each fraction using the automated phosphopeptide enrichment (APE) method described by Tape et al. (2014b). Phosphopeptide fractions were individually desalted using OLIGO R3 resin (Life Technologies 1-1339-03) and resuspended in 0.1% formic acid prior to Q-Exactive Plus HCD FT/FT LC-MS/MS (see the Supplemental Experimental Procedures). For reciprocal phosphoproteomics PSC-PDA co-cultures, 15 mg protein was digested with 150 μg Lys-C (Wako 125-05061) (24 hr) and 150 μg Trypsin (Worthington) (24 hr) using 2 ml FASP. Lyophilized tryptic peptides were re-suspended in 60% MeCN and resolved using a Ultimate 3000 (Dionex) high-performance liquid chromatography fitted with a 10 μm particle size, 7.8 mm ID, and 30 cm TSKgel Amide-80 hydrophilic interaction liquid chromatography (HILIC) column (Tosoh 14459) (McNulty and Annan, 2008) into 24 fractions. Phosphopeptides were enriched from each fraction by APE. Phosphopeptide fractions (n = 192) were individually desalted using OLIGO R3 resin (Life Technologies 1-1339-03) and re-suspended in 0.1% formic acid prior to LTQ Velos HCD FT/FT LC-MS/MS (see the Supplemental Experimental Procedures).

### Multi-axis Phosphoproteomics

For concurrent PDA cell-autonomous and reciprocal phosphoproteomics, 1 × 10^6^ PSCs were plated in a 6-well dish, stimulated with 5 nM SHH-N (C25II) (R&D Systems 464-SH-025/CF) in DMEM + 0.5% FBS, and conditioned media was collected after 48 hr. PDA cells (1 × 10^6^) were cultured without doxycycline (KRAS^WT^), with 1 μg/ml doxycycline (KRAS^G12D^), and with 1 μg/ml doxycycline (KRAS^G12D^) + PSC+SHH conditioned media (biological n = 3) (all in +0.5% dialyzed FBS). One hundred micrograms of each condition was then processed for TMT and APE analysis as described above.

### Cell-Type Labeling with Amino Acid Precursors

*Mycobacterium tuberculosis* (DDC^*M.tub*-KDEL^) (P0A5M4) diaminopimelate decarboxylase (DDC) and *Proteus mirabilis* lysine racemase (Lyr^M37-KDEL^) (M4GGR9) were synthesized by GeneArt. Full details can be found in Tape et al. (2014a). DDC cells were grown in DMEM (-K/-R) (Caisson DMP49) supplemented with 10% (v/v) dialyzed FBS (GIBCO), 0.3 mM L-arginine (Sigma A8094) and 5 mM meso-2,6-diaminopimelate (DAP) (Sigma 07036). Lyr cells were grown in DMEM (-K/-R) supplemented with 10% (v/v) dialyzed FBS, 0.3 mM L-arginine and either 2.5 mM “Medium” D-lysine-4,4,5,5-d4 HCl (C/D/N D-7334) (Delta mass: 4.025107, Delta average mass: 4.0246) or 2.5 mM “Heavy” D-lysine-3,3,4,4,5,5,6,6-d8 2HCl (C/D/N D-6367) (Delta mass: 8.0502136, Delta average mass: 8.04928).

### Heterocellular Multivariate Phosphoproteomics

PDA cells were transfected with DDC^*M.tub*-KDEL^ and grown on 5 mM DAP (“Light”). PSCs were transfected with Lyr^M37-KDEL^ and grown on 2.5 mM D-lysine-3,3,4,4,5,5,6,6-d8 2HCl (“Heavy”). PDA+DDC cells (3 × 10^6^) were cultured in a 10 cm dish ± 1 μg/ml doxycycline, ±10 μg/ml SHH neutralizing monoclonal antibody (mAb) (R&D Systems MAB4641) and ±3 × 10^6^ “Heavy” PSC+Lyr cells (biological triplicates). All cells were grown in DMEM (-K/-R) supplemented with 0.5% (v/v) dialyzed FBS, 0.3 mM L-arginine, 5 mM DAP, and 2.5 mM “Heavy” D-lysine. After 5 days, each condition was lysed in 6 M urea, sonicated, centrifuged to clear cell debris, and protein concentration was determined by BCA. One hundred micrograms of each variable was then processed for TMT and APE analysis as described above.

### Heterocellular Reciprocal Proteomics

To investigate reciprocal regulation of PDA protein abundance, “Heavy” PDA+Lyr^M37-KDEL^ cells were co-cultured with “Light” PSC+DDC^*M.tub*-KDEL^ in the presence of 2.5 mM “Heavy” D-lysine-3,3,4,4,5,5,6,6-d8 and 5 mM “Light” DAP (biological n = 3). For each experiment, a control co-culture of “Medium” PDA+Lyr^M37-KDEL^ cells and “Light” PSC+DDC^*M.tub*-KDEL^ was performed in the presence of either PDA pre-treatment with IGF1R inhibitor (250 nM picropodophyllin [PPP]), AXL inhibitor (500 nM R428), or 20 μg/ml SHH-neutralizing antibody (R&D Systems MAB4641). All co-cultures were performed in +0.5% dialyzed FBS for 72 hr. Co-cultures were lysed in 100 mM Na_2_CO_3_ (pH 11.0), pooled, snap-frozen in liquid nitrogen, treated with Benzonase (Novagen 70746), centrifuged at 40,000 rpm (to resolve membrane-bound proteins from cytosolic proteins), and denatured in 6 M urea 2 M thiourea. Differential changes in cytoplasmic and membrane protein levels were determined using “In-gel digestion” (see the Supplemental Experimental Procedures). To investigate the comparative KRAS^G12D^ cell-autonomous proteome, KRAS^WT^ “Medium” and “Heavy” PDA+Lyr^M37-KDEL^ cells were seeded into 10-cm dishes (biological n = 3) (5 × 10^6^ PDA cells/plate). Doxycycline (1 μg/ml) was then added to the “Heavy” PDA cells (i.e., KRAS^G12D^) and the “Medium” cells were left untreated (i.e., KRAS^WT^) (in +0.5% dialyzed FBS). After 72 hr, cells were lysed as above.

## Author Contributions

C.J.T. conceived the project, performed all proteomic-signaling experiments, and wrote the paper. S.L. performed PSC secretomics. M.D. and G.P. performed PDA mitochondrial flux analysis. K.M.M. and J.D.W. provided LC-MS/MS support. I.C.N., H.S.L., and C.J.M. performed FACS RNA-seq. D.A.L. oversaw the project. C.J. conceived the project, oversaw the project, and wrote the paper.

## Accession Numbers

The accession number for all 617 mass spectrometry proteomic files have been deposited to the ProteomeXchange Consortium (http://www.proteomexchange.org) via the PRIDE partner repository (Vizcaíno et al., 2013): PXD003223.

## Supplemental Information

Document S1. Supplemental Experimental Procedures

Data S1. Proteomic and Phosphoproteomic Data, Related to Figures 1, 2, 3, 4, and 5 This file contains 14 processed data tables and 5 cellular heat maps. Tables are labeled by figure number for simple navigation and cellular heat maps provide a graphical overview of regulated events. Raw mass-spectrometry data can be found via the PRIDE partner repository with the dataset identifier PXD003223.

Document S2. Article plus Supplemental Information
