# Oncogenic KRAS Regulates Tumor Cell Signaling via Stromal Reciprocation

**DOI:** 10.1016/j.cell.2016.05.079

**Published:** 2016-06-16

**Authors:** Christopher J. Tape, Stephanie Ling, Maria Dimitriadi, Kelly M. McMahon, Jonathan D. Worboys, Hui Sun Leong, Ida C. Norrie, Crispin J. Miller, George Poulogiannis, Douglas A. Lauffenburger, Claus Jørgensen

(Cell *165*, 910–920, May 5, 2016)

Our paper demonstrated the cell-autonomous and non-cell-autonomous effects of oncogene signaling in tumor and stromal cells using a proteomic approach. It has come to our attention that Data S1, which summarized our proteomic and phosphoproteomic data, included two sets of errors. In the tab related to Figure 3E, the data were labeled as representing log2-transformed ratios but were erroneously formatted to represent natural ratios. These numbers have now been changed to represent log2-transformed ratios. In the tab related to Figure 5, a copying error from our proteomics software caused the 6H time values to be incorrectly displayed. These values have also now been corrected. The values represented in the corrected version of Data S1 were the ones that had been used in our analyses throughout the paper, so the conclusions and figures in the paper remain unchanged.

