# Supplementary figures and images for: Oncogenic KRAS Regulates Tumor Cell Signaling via Stromal Reciprocation
Source: Cell. 2016 May 5;165(4):910–20. doi: 10.1016/j.cell.2016.03.029 (PMC4868820; doi:10.1016/j.cell.2016.03.029)

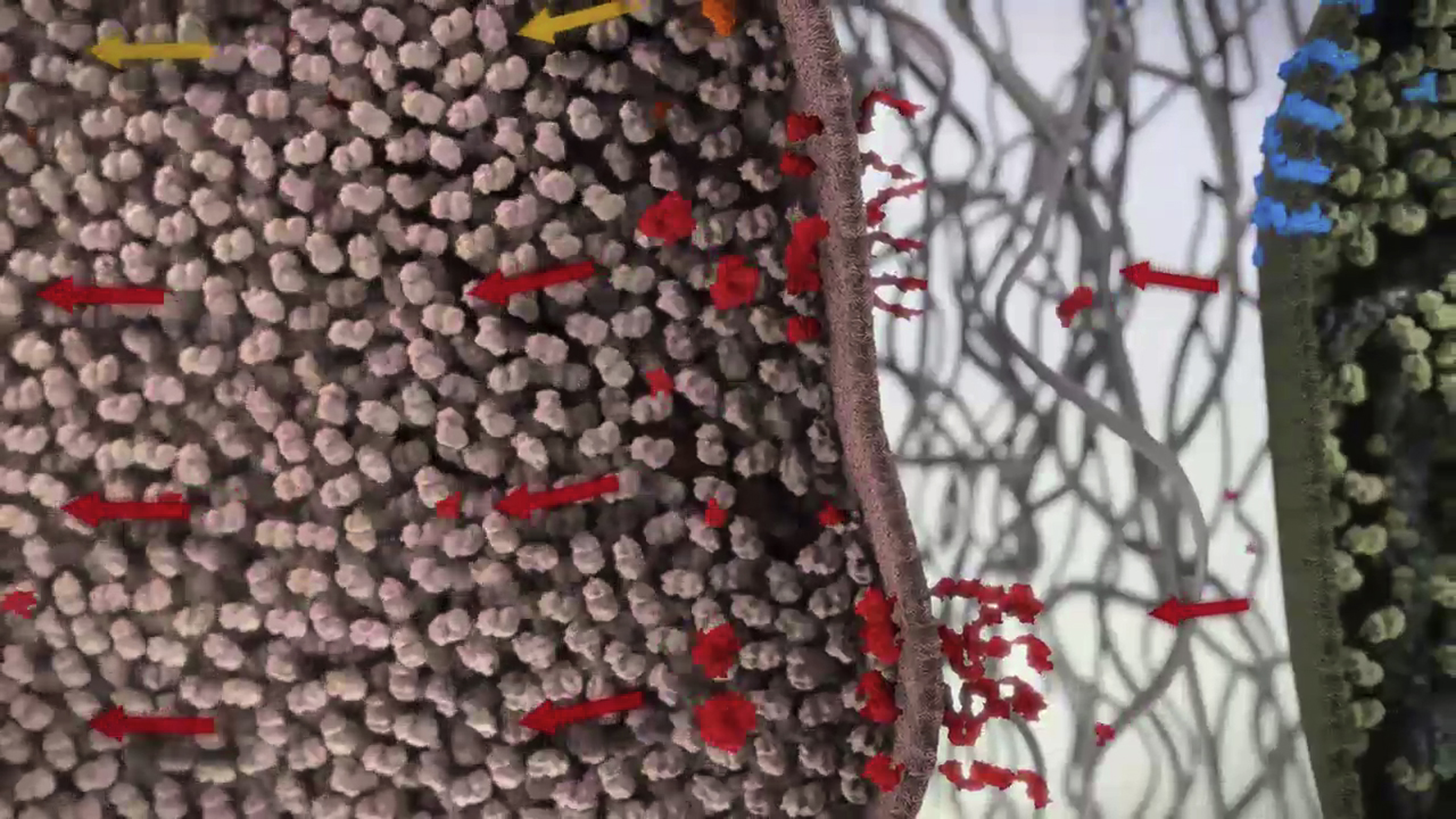

Supplement: Supplementary file 1 [file mmc4.jpg]
