# Supplementary material for: Oncogenic KRAS Regulates Tumor Cell Signaling via Stromal Reciprocation
Source: Cell. 2016 May 5;165(4):910–20. doi: 10.1016/j.cell.2016.03.029 (PMC4868820; doi:10.1016/j.cell.2016.03.029)
Supplement: Document S1. Supplemental Experimental Procedures [file mmc1.pdf]

Cell, Volume *165*

Supplemental Information

Oncogenic KRAS Regulates Tumor Cell Signaling via Stromal Reciprocation

Christopher J. Tape, Stephanie Ling, Maria Dimitriadi, Kelly M. McMahon, Jonathan D. Worboys, Hui Sun Leong, Ida C. Norrie, Crispin J. Miller, George Poulogiannis, Douglas A. Lauffenburger, and Claus Jørgensen

Oncogenic KRAS Regulates Tumor Cell Signaling via Stromal Reciprocation

Christopher J. Tape[1,2], Stephanie Ling[1], Maria Dimitriadi[1,4], Kelly M. McMahon[3], Jonathan D. Worboys[1,3], Hui Sun Leong[3], Ida C. Norrie[1,3], Crispin J. Miller[3], George Poulogiannis[1], Douglas A. Lauffenburger[2], Claus Jørgensen[1,3,*].

[1] The Institute of Cancer Research, 237 Fulham Road, London, SW3 6JB, UK.
[2] Department of Biological Engineering, Massachusetts Institute of Technology, Cambridge, Massachusetts, 02139, USA.
[3] Cancer Research UK Manchester Institute, University of Manchester, Wilmslow Road, Manchester, M20 4BX, UK.
[4] Present address: Department of Biological and Environmental Sciences, University of Hertfordshire, Hatfield, AL10 9AB, UK.
* To whom correspondence should be made: claus.jorgensen@cruk.manchester.ac.uk

SUPPLEMENTAL EXPERIMENTAL PROCEDURES

Cell Lines and Culture

p48-Cre;tetO_LKras^{G12D}; p53$^{fl/+}$ iKRAS PDA cells (1, 2 and 3) (Ying et al., 2012) (a kind gift from Dr. Haoqiang Ying and Dr. Ronald A. DePinho), p48-Cre;R26-rtTa-IRES-EGFP;TetO-Kras^{G12D}; p53$^{fl/+}$ iKRAS cells (A, B and C) (Collins et al., 2012) (a kind gift from Dr. Marina Pasca di Magliano) and mouse PSCs (1, 2 and 3) (Mathison et al., 2010) (a kind gift from Dr. Raul Urrutia) were maintained in DMEM (Gibco 41966052) +10 % (v/v) FBS at 37 ºC, 5 % CO_2. PDA cells were cultured in the presence of 1 µg/mL doxycycline (i.e. KRAS^{G12D}) unless stated otherwise. To remove KRAS^{G12D} (i.e. KRAS^{WT}), cells were cultured without doxycycline for one 24-hour passage prior to each experiment. Loss of KRAS^{G12D} protein-expression was confirmed by western blot (NewEast Bioscience 26036) and observation of the LVVVGADGVGK mutant peptide (Wang et al., 2011) by liquid-chromatography tandem mass-spectrometry (LC-MS/MS) (see below). All experiments were performed in +0.5 % dialyzed FBS (Gibco) to limit serum-induced signaling. To retain cell-surface proteins prior to each co-culture experiment, cells were detached using Enzyme-Free Cell Dissociation Buffer (Life Technologies 13151-014). All cells were PCR screened for (and confirmed clear of) mycoplasma throughout analysis (e-Myco 25233). All cell lines were screened by Li-Cor fluorescent immunoblot for proteins of known importance in PDA. All cells illustrated detectable and comparable levels of PTEN, TGFβR1, SMAD4, c-Raf, MEK1, ERK1/2, AKT, SRC, SMO, SuFu, and Gli1. All activated PSCs expressed αSMA and Vimentin whilst all PDA cells expressed Cytokeratin and E-Cadherin.

PSC SHH-Activated Proteomics

PSCs were SILAC labeled ‘Light’ (K0/R0) and ‘Heavy’ (K8/R10) (described above). All ‘Heavy’ PSCs were treated with 5 nM SHH-N and ‘Light’ PSCs were used as untreated controls. Cells were lysed in 100 mM Na_2CO_3 (pH 11.0), pooled, snap-frozen in liquid nitrogen, treated with Benzonase (Novagen 70746), centrifuged at 40,000 rpm (to resolve membrane-bound proteins from cytosolic proteins) and denatured in 6 M urea 2 M thiourea. Differential changes in cytoplasmic and membrane protein levels were determined using ‘In-gel Digestion’ (detailed below) (biological duplicates). For secretomic analysis, PSCs were labeled ‘Medium’ (K4/R6) and ‘Heavy’ (K8/R10). (Note: ‘Medium’ and ‘Heavy’ SILAC channels were used to distinguish PSC proteins from any residual ‘Light’ serum proteins.) PSCs were seeded at $6x10^6$ PSCs / T75 flask in SILAC DMEM +10% dialyzed FBS, switched to SILAC DMEM +0.5% dialyzed FBS and ‘Heavy’ PSCs were stimulated with 5 nM SHH-N. ‘Medium’ PSCs were used as untreated controls. After 24 hours cells were washed 5 times into serum-free SILAC media and +/- SHH serum-free media was added for another 24 hours. Serum-free conditioned media was collected, pooled, passed through a 0.22 µm filter, and proteins were precipitated using 20% trichloroacetic acid. Differential changes in soluble protein levels were determined using ‘In-gel Digestion’ (see Supplemental Experimental Procedures) (biological duplicates). Relative changes in PSC cytokines and growth factors after 48 hours of SHH-N stimulation were determined using the RayBio® Mouse Cytokine Antibody Array G2000 (RayBiotech AAM-CYT-G2000-8) (144 proteins quantified in duplicate per sample) (biological duplicates / PSC isolation). Growth factor expression was further validated by sandwich ELISA IGF1 (R&D Systems DY791) and GAS6 (R&D Systems DY986).

Primary Cilia Quantification

All cells were cultured as described for the Gli1-Luciferase assay (see below) in a CellCarrier™-96 (PerkinElmer 6005558), fixed with 4% paraformaldehyde, and permeabilized with 0.5% Triton X-100. Cells were blocked with 1% BSA 2% FBS, immunostained for Acetylated Tubulin (Sigma 6-11B-1) (1:5,000) and nuclei (Sigma Hoechst 33258) (1 µg/mL). High-content imaging was performed using the Operetta platform (PerkinElmer) (9 fields/well, 3 wells/cell) and automated quantification was performed using Harmony software (PerkinElmer). Nuclei were used to calculate total cell numbers and primary cilia were quantified using the 'Spots' algorithm. (2000-4000 cells counted per well). % Primary Cilia = (# Acetylated-Tubulin / # Nuclei) x 100.

Gli1-Luciferase Reporter Assay

All cells were transfected (>90 % confluence) with x9Gli1BS-Luciferase reporter (a kind gift from Frederic de Sauvage, Genetech) and CMV-Renilla Luciferase (Pierce 16153) (1:4 DNA:Lipofectamine 2000 (Life Technologies 11668) ratio) in white 96-well plates (Nunc 10072151). Cells were stimulated with 5 nM SHH-N (C25II) (R&D Systems 464-SH-025/CF) in DMEM + 0.5% FBS for 48 hours. 100 nM SANT-1 (Tocris 1974) (Smoothened inhibitor) and 10 µM GANT-61 (Tocris 3191) (Gli inhibitor) were used as intracellular SHH pathway inhibitors. Co-transfection with mouse CMV-Gli1 (Addgene 34996) was used as a positive control for reporter activity in each cell type. Luminescence was quantified using Dual Glo system (Promega E2940). All firefly luciferase results were normalized for transfection efficiency using Renilla luciferase signals. For co-culture assays, PSCs were transfected as above, media was removed, cells were washed and $2x10^4$ PDA cells were added to each well in DMEM + 0.5% FBS (+/- 1 µg/mL doxycycline) for 72 hours. The SHH neutralizing mAb (R&D Systems MAB4641) was used at 10 µg/mL.

SILAC Labeling

All cell lines were grown in K/R-free DMEM (Caisson DMP49) supplemented with 10% dialyzed FBS (Gibco), 100 µg/mL *L*-proline (Bendall et al., 2008) (Sigma P5607) and either 50 µg/mL 0/0 K/R ('Light'), +4 Da/+6 Da K/R ('Medium'), or +8 Da / +10 Da K/R ('Heavy') amino acids (Sigma Isotec). After 5 passages labeling efficiency was >95% for all cell lines (determined by LC-MS/MS, see below). No isotopic arginine > proline conversion was observed.

$KRAS^{G12D}$ Cell-Autonomous Signaling

For immuno-blot analysis, $1x10^6$ $KRAS^{WT}$ PDA cells were plated in a 6-well dish and cultured in DMEM + 0.5% FBS + 1 µg/mL doxycycline. After 2, 4, 6, 8, 12, 24 and 48 hours, cells were lysed in PLC buffer (50 mM HEPES, pH 7.5, 150 mM NaCl, 10% Glycerol, 1% Triton X-100, 1.5 mM $MgCl_2$, 1 mM EGTA), protein concentration was measured by BCA and lysates were Li-Cor fluorescent immuno-blotted for RAS^{G12D} epitope (NewEast Bioscience 26036), RAS (Abcam ab52939), ERK1/2 (pT183 pY185) (Sigma M8159), ERK1/2 (CST 4695), AKT (pS473) (CST 4060), AKT (pT308) (CST 2965), AKT (CST 2920) and β-Actin (Abcam ab8227). For phospho-antibody array analysis, $1x10^6$ $KRAS^{WT}$ PDA cells were plated in a 6-well dish and cultured in DMEM + 0.5% FBS + 1 µg/mL doxycycline. After 0, 4, 8, 12, 24 and 48 hours, cells were lysed, protein concentration was measured by BCA and lysates were analyzed using the PathScan® Intracellular Signaling Array Kit (CST 7744) (18 intracellular signaling nodes in technical

duplicate per sample). Fluorescent intensities were calculated in ‘ImageJ64’ and converted to log_2 fold-difference values (relative to t = 0). Heatmaps (heatmap.2 function, hierarchical clustering) and PCA analysis (prcomp function) were performed in R (version 3.0.1). For multivariate phosphoproteomic analysis, $5x10^6$ $KRAS^{WT}$ PDA cells were plated in a 10 cm dish and cultured in DMEM + 0.5 % FBS, +/- 500 nM AKTi (MK2206) (Selleckchem S1078), +/- 500 nM MEKi (PD 184352) (Tocris 4237), +/- 1 µg/mL doxycycline. After 12 hours each condition was lysed in 6 M urea, sonicated, centrifuged to clear cell debris and protein concentration was determined by BCA. 100 µg of each condition was individually digested by FASP, amine-TMT-10-plex labeled (Pierce 90111) on membrane (iFASP), eluted, pooled, lyophilized and subjected to automated phosphopeptide enrichment (APE) (Tape et al., 2014b). Phosphopeptides were desalted using OLIGO R3 resin (Life Technologies 1-1339-03) and lyophilized prior to LC-MS/MS analysis (see below).

Multi-Axis Phosphoproteomics

For targeted temporal phospho-signaling analysis, $5x10^6$ PSCs were plated in a 10 cm dish, treated +/- 5 nM SHH-N (C25II) (R&D Systems 464-SH-025/CF) in DMEM + 0.5% FBS and conditioned media was collected after 48 hours. $1x10^6$ PDA ($KRAS^{G12D}$) cells were plated in a 6-well dish, serum-starved overnight, and treated with PSC conditioned media +/- SHH. Cells were lysed after 0, 2.5, 5, 7.5, 10, 15, 20, and 30 minutes, protein concentration was measured by BCA and lysates were analyzed using the PathScan® RTK Signaling Array Kit (CST 7949) (28 receptor tyrosine kinases and 11 intracellular signaling nodes in technical duplicate per sample). Fluorescent intensities were calculated in ‘ImageJ64’ and converted to log_2 fold-difference values (relative to t = 0). PCA analysis (prcomp function) was performed in R (version 3.0.1). For multi-axis multivariate perturbation phosphoproteomic analysis, $5x10^6$ $KRAS^{G12D}$ PDA cells were plated in a 10 cm dish and cultured +/- 500 nM AKTi (MK2206) (Selleckchem S1078), +/- 500 nM MEKi (PD 184352) (Tocris 4237) for 30 minutes. Cells were then treated +/- PSC-SHH conditioned media (described above). After 10 minutes each condition was lysed in 6 M urea, sonicated, centrifuged to clear cell debris and protein concentration was determined by BCA. 100 µg of each condition was individually digested by FASP, amine-TMT-10-plex labeled (Pierce 90111) on membrane (iFASP), eluted, pooled, lyophilized, and subjected to automated phosphopeptide enrichment (APE) (Tape et al., 2014b). Phosphopeptides were desalted using OLIGO R3 resin (Life Technologies 1-1339-03) and lyophilized prior to LC-MS/MS analysis (see below). To investigate the dependency of IGF1R and AXL to PDA AKT activation, $1x10^6$ PDA ($KRAS^{G12D}$) cells were plated in a 6-well dish, serum-starved overnight, treated +/- IGF1R inhibitor (250 nM Picropodophyllin (PPP)) and/or AXL inhibitor (500 nM R428), and treated with PSC conditioned media +/- SHH (biological duplicates). Cells were lysed after 5 minutes, protein concentration was measured by BCA and lysates were analyzed using the PathScan® RTK Signaling Array Kit (CST 7949) (28 receptor tyrosine kinases and 11 intracellular signaling nodes in technical duplicate per sample). Fluorescent intensities were calculated in ‘ImageJ64’ and converted to log_2 fold-difference values (relative to t = 0). Heatmap (heatmap.2 function, hierarchical clustering) was performed in R (version 3.0.1). In parallel, 100 µg of each condition was individually digested by FASP, amine-TMT-10-plex labeled (Pierce 90111) on membrane (iFASP), eluted, pooled, lyophilized and subjected to automated phosphopeptide enrichment (APE). Phosphopeptides were desalted using OLIGO R3 resin (Life Technologies 1-1339-03) and lyophilized prior to LC-MS/MS analysis (see below).

Reciprocal Phospho-Signaling From SHH-Activated PSCs To PDA Tumor Cells

Two identical populations of 'Light' (K0/R0) PSCs were seeded on 15 cm plates. One population was activated with 5 nM SHH-N and the other population was left untreated. After 96 hours, 'Medium' (K4/R6) PDA cells were added to the untreated PSCs and 'Heavy' (K8/R10) PDA cells were added to the SHH-activated PSCs ($1x10^7$ PDA cells/plate) for 1, 2, 4, and 6 hours (biological duplicates / time point). To avoid endogenous SHH from PDA cells stimulating PSCs in the vehicle control, these control co-cultures were performed in the presence of 10 µg/mL SHH-neutralizing antibody (R&D Systems MAB4641). Co-cultures were lysed in 6 M urea, sonicated, centrifuged to clear cell debris and protein concentration was determined by BCA. Peptides were prepared by FASP, fractionated by HILIC, and phosphopeptides were isolated by APE.

RNA-Seq Analysis Of PDA Cells In Direct Co-Culture

PDA cells constitutively express GFP(Ying et al., 2012). PSCs were infected with RFP pMSCV-pBabeMCS-IRES-RFP retrovirus (Addgene 33337). PDA+GFP cells were co-cultured with PSC+RFP 20 µg/mL SHH-neutralizing antibody (R&D Systems MAB4641) (biological n = 4) in +0.5% dialyzed FBS for 72 hours. Cells were trypsinized and rapidly resolved using a FACSAria (BD Biosciences) according to their GFP and RFP expression. The mCherry channel was used for optimal separation of the PSC+RFP cells. Analyzes were performed using FlowJo (Tree Star Inc.). PDA+GFP mono-populations were instantly lysed, passed through a QIAshredder and snap-frozen. Indexed PolyA Seq libraries were prepared using 500 ng of total RNA and 12 cycles of amplification by the 'Next Ultra Directional RNA Library Prep Kit' (New England Biolabs E7420S). Libraries were quantified by qPCR using a 'Kapa Library Quantification Kit' for Illumina sequencing platforms (Kapa Biosystems KK4835). Pooled libraries were clustered at 15 pM on the cBot and 2 x 100 bp sequencing was carried out using the High Throughput mode of a HiSeq 2500 using TruSeq SBS Kit v3 chemistry (Illumina). 13~20 millions paired-end reads (101 nt) were obtained from all sequencing libraries and aligned to the mouse reference genome GRCm38/mm10 using the splice alignment software TopHat(Trapnell et al., 2009) (version 2.0.9) with default parameters, except for options specifying library type (fr-firststrand). Over 93% of the reads can be mapped to the reference genome with 72% of the reads aligned uniquely. Gene annotation was taken from Ensembl release 74. The expression levels of 39,179 annotated features were determined by using the featureCounts function from the Bioconductor package Rsubread (Liao et al., 2013) (version 1.13.13) using strand-specific counting mode. The Bioconductor package DESeq (version 1.12.1) was used to identify genes that showed statistically significant variation in expressions levels between the reciprocally active relative to the SHHi samples. First, the data was filtered so that only genes with at least 100 reads across samples were kept. 13,165 genes were retained for differential expression analysis after this step. Read counts were normalized using the estimateSizeFactors function, and variance was modeled by the estimateDispersions function. Differential expression analysis was performed using the negative binomial generalized linear model implemented in the function nbinomTest. Genes with Benjamini-Hochberg adjusted p-value < 0.05 were considered as significantly differentially expressed. We used two different functional enrichment tools to characterise the differentially expressed genes. First, we analyzed the gene lists using the online DAVID (Anders and Huber, 2010; Huang et al., 2007) functional enrichment tools with default options. Second, the gene lists were analyzed with the Bioconductor package GOstats (version 2.26.0) by setting the minimum and maximum gene sets size to 5 and 1000, respectively. False discovery rate

(FDR) was kept below 5% in both cases. All RNA-seq data have been deposited in the GEO database under the accession GSE70351.

High-Content Live-Cell Mitochondrial Imaging

PDA cells were plated at $1x10^4$/well in CellCarrier™-96 (PerkinElmer 6005558), treated +/- 1 µg/mL doxycycline, +/- PSC + SHH conditioned media, +/- PSC + SHH + 20 µg/mL SHH-neutralizing antibody (R&D Systems MAB4641), +/- 500 nM AKTi (MK2206) (Selleckchem S1078), or +/- combined IGF1Ri (250 nM Picropodophyllin) and AXLi (500 nM R428). All cultures were performed in +0.5% dialyzed FBS. After 72 hours cells were stained for mitochondrial markers. MitoTracker (Life Technologies M22426) was used at 5 nM (in serum-free DMEM, 45 minutes), MitoSOX (Life Technologies M36008) was used at 0.5 µM (in HBSS, 10 minutes), and Tetramethylrhodamine, Ethyl Ester, Perchlorate (TMRE) (Life Technologies T-669) was used at 0.1 nM (in HBSS, 15 minutes). Negative controls were performed with 10 µM CCCP for 20 mins prior to staining. Cells were washed with x3 PBS and imaged live using the Operetta platform (PerkinElmer) (23 fields/well, 9 wells/variable) and automated intensity quantification was performed using Harmony software (PerkinElmer).

Mitochondrial Flux Analysis

PDA cells were plated at $1x10^7$ in a T175 and treated +/- PSC + SHH conditioned media for 72 hours. All cultures were performed in +0.5% dialyzed FBS. Cells were re-plated at $1.5x10^4$ / well in the presence of conditioned media (n = 6) and mitochondrial respiration was measured using XF Mito Stress Kit (Seahorse Biosciences 103015-100) on a XF^e96 Analyzer (Seahorse Biosciences). 2 µM Oligomycin, 1 µM FCCP, and 0.5 µM rotenone/antimycin (R/A) were used for all conditions. Cell numbers were normalized using Cyquant (Life Technologies).

Heterocellular Proliferation Assay

PDA cells stably transfected with pCMV-Red Firefly Luciferase (Thermo Scientific 16156) were seeded at $1x10^4$/well in white 96-well plates (Nunc 734-2002), and treated with either 500 nM AKTi (MK2206) (Selleckchem S1078), 20 µg/mL SHH-neutralizing antibody (R&D Systems MAB4641) or combined IGF1Ri (250 nM Picropodophyllin) and AXLi (500 nM R428). $1x10^4$/well PSCs were added to engage reciprocal signaling. All cultures were performed in +0.5% dialyzed FBS. After 72 hours cell-specific tumor cell proliferation was monitored using Bright-Glo™ Luciferase Assay System (Promega E2620). Results are expressed as RLU fold-difference of monoculture controls.

High-Content TUNEL Imaging

PDA cells were plated at $0.5x10^4$/well in CellCarrier™-96 (PerkinElmer 6005558) and treated as described for mitochondrial imaging. After 96 hours cells were fixed with 4% PFA and permeabilized with 0.25% Triton X-100. Cells were then stained using Click-iT TUNEL Alexa Fluor® 647 (Life Technologies C10247) following the manufacture's instructions. A positive control population was treated with DNase I for each variable. Following Hoechst nuclear staining, cells were washed with x3 PBS and imaged using the Operetta platform (PerkinElmer) (23 fields/well, 9 wells/variable) and automated intensity quantification was performed using

Harmony software (PerkinElmer). Hoechst was used to define nuclei and TUNEL intensity was only measured within the nuclear region. Results are presented as a percentage of DNase I TUNEL intensity.

Caspase 3/7 Activity Screening

PDA cells were plated at $0.5x10^4$/well in white 96-well plates (Nunc 10072151), treated +/- 1 μg/mL doxycycline, +/- PSC + SHH conditioned media, +/- PSC + SHH + 20 μg/mL SHH-neutralizing antibody (R&D Systems MAB4641), +/- 100 nM AKTi (MK2206) (Selleckchem S1078), or +/- combined IGF1Ri (250 nM Picropodophyllin) and AXLi (500 nM R428). After 96 hours, cells were analyzed using the ApoTox-Glo™ Triplex Assay (Promega G6321) following the manufacture's instructions. Caspase-3/7 activity was normalized to cell number and presented as RLU fold-difference relative to PSC + SHH conditioned media.

Colony Formation Assay

PDA cells were suspended in base 0.6 % Noble agar (Sigma A5431) (containing DMEM, 0.5 % dialyzed FBS, and penicillin-streptomycin (100 units/mL)) and plated at $2x10^4$/well in 6-well plates. A top layer of 0.3 % noble agar (containing DMEM and 0.5 % dialyzed FBS) was added +/- 1 μg/mL doxycycline, +/- PSC + SHH conditioned media, +/- PSC + SHH + 20 μg/mL SHH-neutralizing antibody (R&D Systems MAB4641), +/- 100 nM AKTi (MK2206) (Selleckchem S1078), or +/- combined IGF1Ri (250 nM Picropodophyllin) and AXLi (500 nM R428). Top layer agar was replaced every 3 days. After 21 days cells were fixed with 4% PFA, washed with PBS, stained with crystal violet 0.5% (in 10 % ethanol), washed with PBS and scanned. Colony area percentage was calculated with ImageJ 'ColonyArea' (Guzman et al., 2014).

In-gel Protein Digestion

For SHH-stimulated PSC experiments, 50μg of 1:1 mixed PSC (K0/R0):PSC+SHH (K8/R10) lysates were resolved on a pre-cast SDS-PAGE gel (Bio-Rad 456-9033) (biological duplicates). Cytosolic and membrane fractions were run separately to increase proteome coverage. 12 gel bands were excised per lane (total gel bands = 48). Proteins were reduced with 10 mM dithiothreitol (50 °C, 1 hour), alkylated with 50 mM iodoacetamide (room temp, 1 hour) and digested with 50 ng/band Trypsin (Promega V5111) (37 °C, 16 hours). Peptides were eluted in 5% trifluoroacetic acid (TFA), 50% MeCN and resuspended in 0.1% TFA prior to CID FT/IT LC-MS/MS (see below). For SHH-activated PSC secretomic experiments, 1:1 mixed PSC (K4/R6):PSC+SHH (K8/R10) protein precipitates were resolved on a pre-cast SDS-PAGE gel (Bio-Rad 456-9033) (biological duplicates). 8 gel bands were excised per lane (total gel bands = 16). Peptides were prepared as described above. For reciprocal regulation of PDA protein abundance, 50 μg of 1:1 mixed co-culture lysates were resolved on a pre-cast SDS-PAGE gel (Bio-Rad 456-9033) (biological *n* = 3) for all conditions. Cytosolic and membrane fractions were run separately to increase proteome coverage (24 gel bands per replicate condition). Peptides were prepared as described above. For $KRAS^{G12D}$ cell-autonomous regulation of PDA protein abundance 50 μg of 1:1 mixed mono-culture lysates were resolved on a pre-cast SDS-PAGE gel (Bio-Rad 456-9033) (biological *n* = 3) and peptides were prepared as described above.

Data-Dependent Acquisition (DDA) LC-MS/MS

SILAC samples were run on a LTQ Orbitrap Velos mass spectrometer (Thermo Scientific) coupled to a NanoLC-Ultra 2D (Eksigent). Reverse-phase chromatographic separation was performed on a 100 μm i.d. x 20 mm trap column packed in house with C18 (5 μm bead size, Reprosil-Gold, Dr Maisch), a 75 μm i.d. x 30 cm column packed in house with C18 (5 μm bead size, Reprosil-Gold, Dr Maisch) using a 120 minute linear gradient of 0-50% solvent B (MeCN 100% + 0.1% formic acid (FA)) against solvent A (H_2O 100% + 0.1% FA) with a flow rate of 300 nL/min. The mass spectrometer was operated in the data-dependent mode to automatically switch between Orbitrap MS and MS/MS acquisition. Survey full scan MS spectra (from m/z 375-2000) were acquired in the Orbitrap with a resolution of 60,000 at m/z 400 and FT target value of 1 x 10^6 ions. The 20 most abundant ions were selected for fragmentation using collision-induced dissociation (CID) and dynamically excluded for 8 seconds. For phosphopeptide samples the 10 most abundant ions were selected for fragmentation using higher-energy collisional dissociation (HCD) and scanned in the Orbitrap at a resolution of 7,500 at m/z 400. Selected ions were dynamically excluded for 8 seconds. For accurate mass measurement, the lock mass option was enabled using the polydimethylcyclosiloxane ion (m/z 445.120025) as an internal calibrant. CTAP (Tape et al., 2014a) and TMT samples were run on a Q-Exactive Plus mass spectrometer (Thermo Scientific) coupled to a Dionex Ultimate 3000 RSLC nano system (Thermo Scientific). Reversed-phase chromatographic separation was performed on a C18 PepMap 300 Å trap cartridge (0.3 mm i.d. x 5 mm, 5 µm bead size; loaded in a bi-directional manner), a 75 µm i.d. x 50 cm column (5 µm bead size) using a 120 minute linear gradient of 0-50% solvent B (MeCN 100% + 0.1% formic acid (FA)) against solvent A (H_2O 100% + 0.1% FA) with a flow rate of 300 nL/min. The mass spectrometer was operated in the data-dependent mode to automatically switch between Orbitrap MS and MS/MS acquisition. Survey full scan MS spectra (from m/z 400-2000) were acquired in the Orbitrap with a resolution of 70,000 at m/z 400 and FT target value of 1 x 10^6 ions. The 20 most abundant ions were selected for fragmentation using higher-energy collisional dissociation (HCD) and dynamically excluded for 30 seconds. Fragmented ions were scanned in the Orbitrap at a resolution of 17,500 (CTAP) or 35,000 (TMT) at m/z 400. For TMT samples the isolation window was reduced to 1.2 m/z and a MS/MS fixed first mass of 120 m/z was used. For accurate mass measurement, the lock mass option was enabled using the polydimethylcyclosiloxane ion (m/z 445.120025) as an internal calibrant. For peptide identification, raw data files produced in Xcalibur 2.1 (Thermo Scientific) were processed in Proteome Discoverer 1.4 (Thermo Scientific) and searched against SwissProt mouse (2011_03 release, 15,082,690 entries) database using Mascot (v2.2). Searches were performed with a precursor mass tolerance set to 10 ppm, fragment mass tolerance set to 0.05 Da and a maximum number of missed cleavages set to 2. Static modifications was limited to carbamidomethylation of cysteine, and variable modifications used were oxidation of methionine, deamidation of asparagine / glutamine, isotopomeric labeled lysine (+4.025107 Da and +8.014199 Da), isotopomeric labeled arginine (+6.020129 Da and +10.008269 Da) and phosphorylation of serine, threonine and tyrosine residues. For heterocellular multivariate analysis custom CTAP+TMT10 lysine (+237.213146 Da) and SILAC+TMT10 lysine (+237.177131 Da) modifications were used to identify lysine-heavy residues bound to amine-reactive TMT. Peptides were further filtered using a mascot significance threshold <0.05, a peptide ion Score >20 and a FDR <0.01 (evaluated by Percolator (Kall et al., 2007)). Phospho-site localization probabilities were calculated with phospho*RS* 3.1 (>75%, maximum 4-PTM/peptide) (Taus et al., 2011). Only lysine containing peptides were included in the CTAP quantitative analysis. All 617 mass spectrometry proteomic files have been deposited to the ProteomeXchange Consortium (http://www.proteomexchange.org) via the

PRIDE partner repository (PMID (Vizcaino et al., 2013)) with the dataset identifier PXD003223. Processed data and illustrated cellular heat maps can be viewed in Data S1.

Phosphoproteomic Data Analysis

Phosphopeptides from Proteome Discoverer 1.4 were normalized against total protein levels (from in-gel digest experiments), and protein-level phospho-site locations (phospho*RS* 3.1 score >75%, maximum 4-PTM/peptide) were manually annotated using PhosphoSitePlus. Phosphoproteomic volcano plots display mean Proteome Discoverer 1.4 quantification fold-difference values across all replicates ($\log_2$) against two-tailed t-test P values (calculated from arrays of raw MS/MS TMT intensity counts). Volcano plots were assembled in GraphPad Prism 6 (non-linear Gaussian regression, least squares fit). For principle component analysis (PCA), Proteome Discoverer 1.4 quantification ratio values were converted to $\log_2$, imported into R (version 3.0.1), computed using the function 'princomp(X)' and plotted in GraphPad Prism 6. Empirical parent kinases were manually identified by referenced Uniprot annotation and putative parent kinases were manually assigned using ScanSite (Obenauer et al., 2003) 3 ('High-Stringency' setting, top 0.2% of all sites, lowest score). Phospho-sites that did not meet these conditions were not annotated. Cellular protein location was assigned by Uniprot annotation. Data-driven network illustrations were compiled in OmniGraffle Professional 5. All phosphopeptides were aligned using 'Protein Modification Toolkit' (http://ms.imp.ac.at/?goto=pmt), submitted to Motif-X (Schwartz and Gygi, 2005) (significance 0.000001, occurrences = 20) (foreground = regulated population ($\log_2 > 1$, $P < 0.01$ (when replicates available)), background = non-regulated) and displayed using WebLogo (Crooks et al., 2004).

REFERENCES

Anders, S., and Huber, W. (2010). Differential expression analysis for sequence count data. Genome Biol *11*, R106.

Bendall, S.C., Hughes, C., Stewart, M.H., Doble, B., Bhatia, M., and Lajoie, G.A. (2008). Prevention of amino acid conversion in SILAC experiments with embryonic stem cells. Mol Cell Proteomics *7*, 1587-1597.

Collins, M.A., Bednar, F., Zhang, Y., Brisset, J.C., Galban, S., Galban, C.J., Rakshit, S., Flannagan, K.S., Adsay, N.V., and Pasca di Magliano, M. (2012). Oncogenic Kras is required for both the initiation and maintenance of pancreatic cancer in mice. The Journal of clinical investigation *122*, 639-653.

Crooks, G.E., Hon, G., Chandonia, J.M., and Brenner, S.E. (2004). WebLogo: a sequence logo generator. Genome research *14*, 1188-1190.

Guzman, C., Bagga, M., Kaur, A., Westermarck, J., and Abankwa, D. (2014). ColonyArea: an ImageJ plugin to automatically quantify colony formation in clonogenic assays. PLoS One *9*, e92444.

Huang, D.W., Sherman, B.T., Tan, Q., Kir, J., Liu, D., Bryant, D., Guo, Y., Stephens, R., Baseler, M.W., Lane, H.C.*, et al.* (2007). DAVID Bioinformatics Resources: expanded annotation database and novel algorithms to better extract biology from large gene lists. Nucleic Acids Res *35*, W169-175.

Kall, L., Canterbury, J.D., Weston, J., Noble, W.S., and MacCoss, M.J. (2007). Semi-supervised learning for peptide identification from shotgun proteomics datasets. Nature methods *4*, 923-925.

Liao, Y., Smyth, G.K., and Shi, W. (2013). The Subread aligner: fast, accurate and scalable read mapping by seed-and-vote. Nucleic Acids Res *41*, e108.

Mathison, A., Liebl, A., Bharucha, J., Mukhopadhyay, D., Lomberk, G., Shah, V., and Urrutia, R. (2010). Pancreatic stellate cell models for transcriptional studies of desmoplasia-associated genes. Pancreatology *10*, 505-516.

Obenauer, J.C., Cantley, L.C., and Yaffe, M.B. (2003). Scansite 2.0: Proteome-wide prediction of cell signaling interactions using short sequence motifs. Nucleic Acids Res *31*, 3635-3641.

Schwartz, D., and Gygi, S.P. (2005). An iterative statistical approach to the identification of protein phosphorylation motifs from large-scale data sets. Nature biotechnology *23*, 1391-1398.

Tape, C.J., Norrie, I.C., Worboys, J.D., Lim, L., Lauffenburger, D.A., and Jorgensen, C. (2014a). Cell-specific labeling enzymes for analysis of cell-cell communication in continuous co-culture. Mol Cell Proteomics *13*, 1866-1876.

Tape, C.J., Worboys, J.D., Sinclair, J., Gourlay, R., Vogt, J., McMahon, K.M., Trost, M., Lauffenburger, D.A., Lamont, D.J., and Jorgensen, C. (2014b). Reproducible Automated Phosphopeptide Enrichment Using Magnetic TiO2 and Ti-IMAC. Anal Chem *86*, 10296-10302.

Taus, T., Kocher, T., Pichler, P., Paschke, C., Schmidt, A., Henrich, C., and Mechtler, K. (2011). Universal and confident phosphorylation site localization using phosphoRS. Journal of proteome research *10*, 5354-5362.

Trapnell, C., Pachter, L., and Salzberg, S.L. (2009). TopHat: discovering splice junctions with RNA-Seq. Bioinformatics *25*, 1105-1111.

Vizcaino, J.A., Cote, R.G., Csordas, A., Dianes, J.A., Fabregat, A., Foster, J.M., Griss, J., Alpi, E., Birim, M., Contell, J.*, et al.* (2013). The PRoteomics IDEntifications (PRIDE) database and associated tools: status in 2013. Nucleic Acids Res *41*, D1063-1069.

Wang, Q., Chaerkady, R., Wu, J., Hwang, H.J., Papadopoulos, N., Kopelovich, L., Maitra, A., Matthaei, H., Eshleman, J.R., Hruban, R.H.*, et al.* (2011). Mutant proteins as cancer-specific biomarkers. Proceedings of the National Academy of Sciences of the United States of America *108*, 2444-2449.

Ying, H., Kimmelman, A.C., Lyssiotis, C.A., Hua, S., Chu, G.C., Fletcher-Sananikone, E., Locasale, J.W., Son, J., Zhang, H., Coloff, J.L.*, et al.* (2012). Oncogenic Kras maintains pancreatic tumors through regulation of anabolic glucose metabolism. Cell *149*, 656-670.
